# Delving Into the Depths of *AGTR2*: In Silico Identification of Deleterious Nonsynonymous SNPs Associated With Cardiovascular Diseases

**DOI:** 10.1155/humu/9394808

**Published:** 2026-04-15

**Authors:** Muhammad Waleed Iqbal, Muhammad Shahab, Xinxiao Sun, Shahina Akter, Guojun Zheng, Gamal A. Shazly, Mohammed Bourhia, Musaab Dauelbait, Qipeng Yuan

**Affiliations:** ^1^ College of Life Science and Technology, Beijing University of Chemical Technology, Beijing, China, buct.edu.cn; ^2^ Genomic Research Laboratory Bangladesh Council of Scientific and Industrial Research (BCSIR), Dhaka, Bangladesh; ^3^ Department of Pharmaceutics, College of Pharmacy, King Saud University, Riyadh, Saudi Arabia, ksu.edu.sa; ^4^ Laboratory of Biotechnology and Natural Resources Valorization, Faculty of Sciences, Ibn Zohr University, Agadir, Morocco, uiz.ac.ma; ^5^ Department of Scientific Translation, Faculty of Translation, University of Bahri, Khartoum, Sudan, bahri.edu.sd

**Keywords:** *AGTR2*, cardiovascular diseases, in silico analysis, MD simulation, nsSNPs

## Abstract

**Background and Aim:**

Nonsynonymous single nucleotide polymorphisms (nsSNPs) in angiotensin Type II receptor (*AGTR2*) have been identified as a potential cause of cardiovascular illness in humans. Identifying structurally and functionally relevant alterations in *AGTR2* is critical to investigate possible therapeutic targets.

**Methods:**

A comprehensive computational pipeline was employed to evaluate deleterious nsSNPs using multiple prediction algorithms, including SIFT, PolyPhen‐2, CADD, REVEL, Mutation Assessor, MetaLR, I‐Mutant, MutPred, and Phylo3D. Molecular docking and molecular dynamic simulation strategies were further utilized to thoroughly validate these nsSNPs. Additionally, gene–gene interaction networks were constructed to explore *AGTR2*′s functional associations.

**Results:**

Our findings indicated that four nsSNPs, including rs200599388, rs1556673810, rs3729979, and rs1556673736, potentially have the most deleterious effect on the *AGTR2* gene. MD simulations revealed that these variants induced increased structural fluctuations and conformational instability compared with the wild‐type protein. Gene–gene interaction analysis indicated that *AGTR2* participates in several key regulatory pathways relevant to cardiovascular physiology.

**Conclusion:**

These findings will form the basis to design precision medicines for cardiovascular diseases in the future and welcome further preclinical and clinical investigations.

## 1. Introduction

Human genetic variation is primarily attributed to single nucleotide polymorphisms (SNPs), which are the most common type of genetic mutations and thought to play a role in individual differences in physical characteristics. Determining the SNPs responsible for these particular features is a challenging task [1]. Among these mutations, nonsynonymous single nucleotide polymorphisms (nsSNPs) are the most deleterious that alter the amino acid sequence of the protein they encode and are discovered in the coding regions of genes [2]. Because of their direct correlation with alterations in proteins, nsSNPs are thought to be the most significant element among all varieties in the human genome when it comes to heritable human disorders. For example, in the Human Gene Mutation Database (HGMD), nsSNPs account for around half of the genetic variants that cause disease [3]. Because the nsSNPs are involved in the crucial amino acid changes that could have a negative or neutral effect on protein structure or function, a significant effort was applied to delve deeper into their damaging effects [4].

As nsSNPs have been found to contribute for about half of the genetic diseases, numerous investigations have also demonstrated their involvement in cardiovascular disease, which are the main factor of mortality in both industrialized and developing nations [5]. In cardiovascular diseases, several risk factors including genetics have been reported to contribute to their complex etiology [6]. In this regard, angiotensin II is crucial as it is a key component of the renin–angiotensin system (RAS), which is vital in maintaining cardiovascular functioning and blood pressure homeostasis [7]. The primary mechanisms by which angiotensin II acts on the cardiovascular system are through the angiotensin II receptor Types 1 (*AGTR1*) and 2 (*AGTR2*) [8, 9]. According to several studies, *AGTR2* mutations may affect the RAS′s protective benefits by compromising its functionality. Unopposed ATR2 signaling may arise from this, which may cause inflammation, vasoconstriction, and cardiovascular diseases [10]. Variations in the *AGTR2* gene have been linked in various studies to an increased risk of heart failure, coronary artery disease, and hypertension [11, 12]. A crucial type of cardiovascular illness that continues to be a major global public health concern is coronary heart disease (CHD) [13, 14], which is also caused and progressed by ATR2 genetic abnormalities. Furthermore, important data have been produced by *AGTR2* gene disruptions in animal models as well. An animal study demonstrated that mice lacking functional AT2R exhibit increased blood pressure, cardiac hypertrophy, and compromised heart function, suggesting that *AGTR2* could have a protective effect on the cardiovascular system [15].

Globally, cardiovascular problems rank among the most common medical conditions. They account for a sizable portion of mortality and are the primary cause of death globally [16]. Therefore, it is essential to look into each aspect of the cause. In the current research, we examined nsSNPs in the *AGTR2* gene and found that their effects on protein structure and function were detrimental. Several bioinformatics techniques were used to identify the *AGTR2* protein′s most harmful nsSNPs. We created a three‐dimensional model of the wild‐type *AGTR2* protein along with the likely harmful nsSNPs variation. The proposed nsSNPs were comprehensively validated by docking and molecular dynamic (MD) simulation analyses. Overall, this work represents the first of its kind to investigate *AGTR2* proteins in silico, which may prove useful in the management of diseases brought on by nsSNPs. Further clinical and preclinical applications will boost up our conclusion, which may be helpful against cardiovascular diseases.

## 2. Methodology

**Figure 1:**
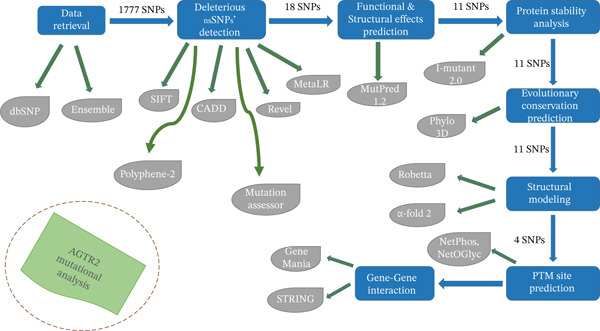
A workflow presenting steps to detect most damaging nsSNPs.

The schematic flow of the entire procedure is shown in Figure 1. Every tool and web server employed in this study, used GRCh38 as the human genome reference [17].

### 2.1. Retrieving nsSNPs

The National Center for Biotechnology Information (NCBI) database for SNPs, dbSNP (https://www.ncbi.nlm.nih.gov/snp/) [18], was utilized to retrieve all *AGTR2*‐related SNPs along with relevant data, including their locations and global minor allele frequencies (MAFs). A total of 1777 SNPs were initially acquired from which 240 nsSNPs were selected based on their potential to induce structural and functional alterations [19]. These nsSNPs were further cross‐validated with the Ensembl database (https://www.ensembl.org/) [20], resulting in the shortlisting of 212 nsSNPs for further analysis.

### 2.2. Deleterious nsSNPs Detection

Six bioinformatics tools were applied to identify the deleterious nsSNPs from all the retrieved SNPs. Among these web‐based tools were SIFT (https://sift.bii.a-star.edu.sg/) [21, 22], Polyphene‐2 (http://genetics.bwh.harvard.edu/pph2/) [23], CADD (https://cadd.gs.washington.edu/) [24], Revel (https://sites.google.com/site/revelgenomics/) [25], MetaLR (http://sites.google.com/site/jpopgen/dbNSFP) [26], and Mutation Assessor (http://mutationassessor.org/r3/) [27]. Subsequent processing was performed on the identified harmful and intolerant nsSNPs from all six tools.

### 2.3. Prediction of Functional and Structural Effects

The structural and functional consequences of the nsSNPs were estimated by employing a web‐based tool, MutPred 1.2 (http://mutpred.mutdb.org/) [28]. This web‐based tool estimates the origin of diseases and finds alternatives for amino acids. A number of structural and functional characteristics, including helical propensity gain and phosphorylation site loss, are screened by this technique. We submitted the *AGTR2* protein sequence in FASTA format and found harmful nsSNPs, which showed abnormal confidence in the form of *p* values. Normal confidence was indicated by *p* < 0.05 and high confidence by *p* < 0.01.

### 2.4. Analysis of Protein Stability

We employed I‐Mutant 2.0 (http://folding.biofo http://ld.org/i-mutant/i-mutant-2.0.html), a web‐server built on the support vector machine (SVM) algorithm [29], to evaluate the stability of our target protein. With RI (dependability index) values ranging from 0 to 10, representing the lowest and highest dependability, respectively, it forecasts changes in the stability of mutant proteins. In order to predict the impact of harmful nsSNPs on the *AGTR2* protein, the protein sequence was exposed to a threshold of pH 7.0 and a temperature of 25°C.

### 2.5. Estimation of Protein Phylogeny Conservation

Each amino acid in the *AGTR2* protein sequence was evaluated for evolutionary conservation using the Phylo3D program (https://github.com/logogin/phylo3D) [30]. Phylo3D analysis focuses on evolutionary interactions between homologous sequences. Using 50 different homologous sequences, the degree of conservation of amino acid residues was estimated. Highly conserved residues that matched to damaging nsSNPs were further studied.

### 2.6. Selection of Most Deleterious nsSNPs

The *AGTR2* gene crystal structure was previously recorded in Protein Data Bank (PDB) (https://www.rcsb.org/) under the pdb id of 5UNF. Three‐dimensional models of 12 mutants associated with the most harmful nsSNPs were created using Robetta (https://robetta.bakerlab.org/) [31]. Using the Rosetta suite, Robetta is a 3D homology modeling program that generates 3D models for proteins. Using TM‐align (https://zhanggroup.org/TM-align/), wild‐type *AGTR2* was compared with selected mutants. It normally forecasts the structural superposition, root mean square deviation (RMSD), and TM‐score. The range of TM scores is 0 to 1, where a score of 1 denotes more structural similarity. Diversity between mutant and wild‐type structures increases with larger RMSD values [32]. Moreover, the ClinVar database (https://www.ncbi.nlm.nih.gov/clinvar/) was utilized to check the clinical significance of genetic variants, which uses already submitted case reports and published research to give results [33].

### 2.7. Secondary Structure Analysis

Secondary structure analysis can offer useful information on a protein′s local structural properties, such as the existence of alpha‐helices, beta strands, and coil regions. This information is critical for understanding the protein′s overall three‐dimensional structure and function [34]. We evaluated the secondary structure of *AGTR2* with an online program, PSIPRED (Protein Structure Prediction) (http://bioinf.cs.ucl.ac.uk/psipred/) [35]. To delve deeper into mutant stability, we used a web‐based program called pCysMod (http://pcysmod.omicsbio.info) [36]. It is a sophisticated web server that predicts many forms of cysteine changes.

### 2.8. Tertiary Structure Prediction

The proposed highly deleterious mutants were, then, submitted to the AlphaFold2 (https://alphafold.ebi.ac.uk/) for further investigation of protein 3D structure comparisons. The resultant protein structure was shown interactively using Pymol visualization software [37], and its molecular properties were examined. After having the modeled 3D structures for all the four mutants, we utilized QMEAN (https://swissmodel.expasy.org/qmean/) [38], MolProbity (http://molprobity.biochem.duke.edu/) [39], and SAVES server (https://saves.mbi.ucla.edu/) [40] to further validate them.

### 2.9. Comprehensive Docking Analysis

We used computational docking to delve deeper into the possible interactions of wild‐type *AGTR2* and its mutants with angiotensin‐converting enzyme 2 (ACE2). ACE2 is an important factor in the RAS, acting as a counter‐regulatory enzyme to *AGTR2* [41]. Furthermore, the interaction of *AGTR2* and ACE2 is known to affect a variety of physiological processes, such as blood pressure control and cardiovascular homeostasis [42–44]. That is why, we chose ACE2 as the receptor with which to bind *AGTR2*. For this aim, we obtained the 3D structure of the ACE2 protein from PDB (PDB ID: IR42) [45]. As a predocking step, polar hydrogens were introduced into the structure, and its energy was reduced using the Molecular Operation System (MOE) visualization tool [46]. Next, we used the excellent online ClusPro v2.0 docking server (https://cluspro.bu.edu/), which predicts protein interactions using energy calculations [47]. Moreover, HADDOCK (https://www.bonvinlab.org/software/haddock2.2/), a versatile and widely‐used flexible protein–protein docking tool, was also employed to dock all the mutants with ACE2 receptor [48]. From this, we were able to accurately dock the ACE2 and *AGTR2* mutants after modeling their probable interactions. Finally, we used the PDBsum server to investigate the specifics of these relationships [49]. The exact amino acid residues, bonds, and forces revealed by this technology help us better understand the various paths of communication between *AGTR2* and cardiovascular disorders.

### 2.10. MD Simulation

MD simulation has emerged as one of the most reliable computational metrics to evaluate the binding potential, structural stability, and conformational changes within mutant–receptor interactions [50]. All the proposed mutants (including I132F, I135N, W269C, and P271L), along with the wild‐type *AGTR2*, were assessed for their atomic behavior while bounded to ACE2 receptor for 200 ns. A general AMBER force field (ff19SB) was calibrated within its Tleap module to manage the system preparation, fixing bonds, and angles. Na^+^ and Cl^−^ ions were added to neutralize the system and a water box was included to surround the prepared system [51]. A two‐step energy minimization protocol was performed to remove structural clashes and fix bad dihedral angles within each system [52]. In the first stage, a conjugate gradient (1500 steps) was applied with an overall 2500 steps, followed by a gradient descent (1000 steps) approach on a constrained protein–protein system. In the later stage, the constraints were lifted and the complex was exposed to complete energy minimization with the same protocol of 2500 steps [53]. Subsequently, each minimized system was heated up to 300 K, followed by the equilibration for 1000 ps. A Berendsen barostat [54] was applied to balance the system pressure, in support with a Langevin thermostat [55] to regulate temperature. The SHAKE algorithm was also employed to strengthen the covalent bonding between residues. Finally, a production run of 200 ns was carried out on each complex to assess their dynamic behavior. The simulations were run using the GPU version of AMBER22 (i.e., PMEMD.cuda). The resulted trajectories were analyzed using state‐of‐the‐art postsimulation analyses (details are in 3.10).

### 2.11. Posttranscriptional Modification Site Prediction

Protein function can be predicted by studying their PTMs. GPS‐MSP 1.0 (https://msp.biocuckoo.org/) was used to predict methylation sites in FUT2. GPS 3.0 (http://gps.biocuckoo.org/online.php) and NetPhos 3.1 (https://services.healthtech.dtu.dk/services/NetPhos-3.1/) were used to predict serine, tyrosine, and threonine phosphorylation sites in the *AGTR2* protein sequence. GPS 3.0 predicted more specific outcomes with a higher likelihood of phosphorylation than NetPhos 3.1 [56], whereas NetPhos 3.1 used neural network ensembles with a threshold of 0.5 [57]. It was anticipated that residues scoring higher than this threshold would be phosphorylated. The *AGTR2* protein′s ubiquitylation sites were predicted using RUBI (http://old.protein.bio.unipd.it/rubi/). RUBI utilized a balanced cutoff and predicted ubiquitination for lysine residues [58]. Glycosylation is an important PTM event that was predicted using NetOglyc4.0 (https://services.healthtech.dtu.dk/services/NetOGlyc-4.0) [59]. Protein sequences having substitutions in amino acids as well as wild‐type sequences are analyzed by this online service. A functionally significant mutation indicates the mutant′s functional pattern deviation from the wild‐type pattern.

### 2.12. *AGTR2* Gene–Gene Interaction

The *AGTR2* gene′s interactions with other genes and the potential effects of nsSNPs on related genes were investigated using GeneMANIA (https://genemania.org/) [60] and STRING (https://string‐db.org/cgi) [61]. Based on coexpression, pathways, colocalization, protein domain similarity, and genetic/protein interaction, GeneMANIA predicts gene–gene relationships. Based on factors including gene fusions, co‐occurrence, coexpression, and experimental and biochemical data, STRING identified the Top 10 most interaction genes. The aggregate score for each gene interacting with the target gene varied from 0 to 1, with 0 representing the lowest interaction and 1 representing the strongest interaction. Using *AGTR2* as the input gene, a network of gene–gene interactions was established.

## 3. Results

### 3.1. Retrieved nsSNPs

**Figure 2:**
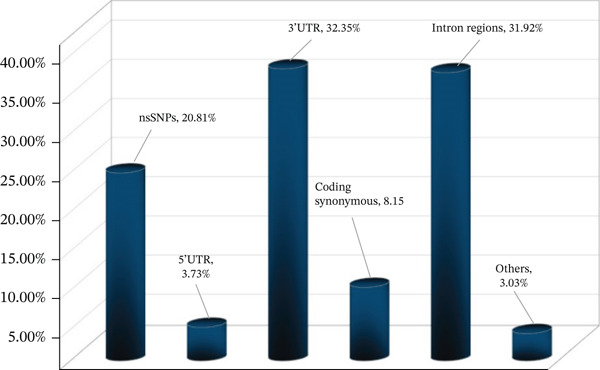
A graph representing the percentage of each category of SNPs related to the *AGTR2* gene.

Two hundred forty nsSNPs, 43 in the 5 ^′^UTR, 373 in the 3 ^′^UTR, 94 coding synonymous, 368 in the intron region, and the remaining SNPs (splice sites = 2, frameshift = 21, nonsense = 12) were extracted from dbSNP, the largest SNP database. A total of 1777 SNPs were obtained from this database, from which only missense nsSNPs were processed further. Ensemble was also utilized to confirm these missense SNPs, resulting in the consensus 212 nsSNPs. Once duplicates have been eliminated, these nsSNPs are manually screened. An illustration of retrieved SNPs is shown in Figure 2.

### 3.2. Prediction of Deleterious nsSNPs

**Figure 3:**
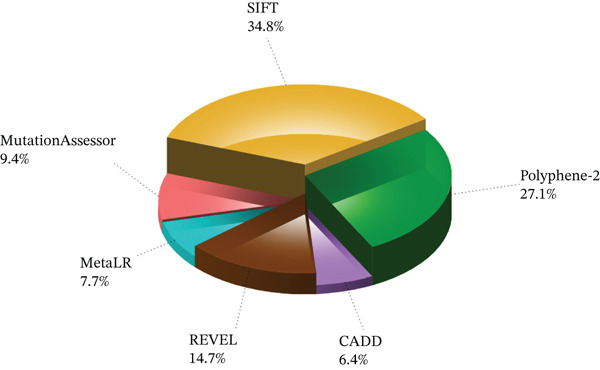
Percentage of deleterious SNPs, predicted by SIFT, Polyphene‐2, MetaLR, Revel, Mutation Assessor, and CADD.

Six distinct bioinformatics tools including SIFT, Polyphene‐2, CADD, Revel, MetaLR, and Mutation Assessor, were applied to all the shortlisted nsSNPs in order to assess their potential impact on the structure and function of the *AGTR2* protein. SIFT refers to replacements as “deleterious” if the score (TI = Total Index) is less than 0.05, and “tolerated” if it is greater than 0.05 [21, 22]. SIFT results demonstrated that 104 nsSNPs had negative effects. The PolyPhen‐2 score indicates the probability of damage from a replacement; values nearer one indicate a stronger expectation of harm [23]. Eighty‐one nsSNPs were predicted by Polyphene‐2 to be possibly deleterious. Similarly, CADD identifies an SNP as detrimental or benign based on its prediction score (e.g., a high score predicts deletion and vice versa) [24]. Nineteen nsSNPs were shown to be possibly disease‐causing by CADD. Higher score mutations are anticipated to be more pathogenic in Revel; scores range from 0 to 1 [25]. Following meticulous prediction, 44 nsSNPs were determined to be ill. Since MetaLR assigns a score between 0 and 1, greater values denoting a higher likelihood of injury, it was also employed. According to the MetaLR evaluation, 23 variations were found to be deleterious. Lastly, Mutation Assessor was used to evaluate variants, which found 28 mutations as extremely damaging. All of these findings are displayed in Figure 3 For the final selection, we prioritized nsSNPs that consistently scored as deleterious across multiple tools. As all the mentioned tools have their limitations, we did not rely on only one of them. Instead, we considered the results of all these tools and shortlisted 18 nsSNPs that were found to be consensus and predicted deleterious across all the six mentioned tools, for further analysis.

### 3.3. Prediction of Structural and Functional Changes

The chosen 18 nsSNPs were analyzed using the MutPred server. The predicted functional and structural effects included modifications to the transmembrane protein, ordered interface, catalytic site, relative solvent accessibility, allosteric site, GPI‐anchor amidation, N‐linked glycosylation, metal binding, and strand. Data on the mentioned attributes, including *p* values, are included in Table 1 As a threshold of above 0.6 is set, 11 of the 18 nsSNPs were predicted to have an impact on the structure or function of proteins, which underwent further research.

**Table 1:** MutPred 1.2 probability values of damaging SNPs, which are detected in the *AGTR2* gene.

Mutation	*p*
P271L	0.861
I132F	0.653
M138T	0.635
W269S	0.918
V146A	0.555
R251C	0.396
R251H	0.316
F220Y	0.585
Y103C	0.873
Y82N	0.837
V65M	0.468
V262A	0.61
S139N	0.634
W110G	0.937
P48H	0.455
G74R	0.417
I135N	0.816
W269C	0.928

### 3.4. Stability Assessment for *AGTR2*

The *AGTR2* protein′s stability for 11 nsSNPs with amino acid alterations was predicted using I‐Mutant. As shown in Table 2, each of the selected nsSNPs was dealt with individually, and stability was assessed using RI ranging from 0 to 10. Each nsSNP that was evaluated showed a decline in stability. These findings indicated that these 11 nsSNPs would be more detrimental to the *AGTR2* protein by decreasing its stability.

**Table 2:** I‐Mutant stability prediction results for *AGTR2.*

SNP ID	Mutation	Stability
rs3729979	P271L	Decrease
rs200599388	I132F	Decrease
rs200855214	M138T	Decrease
rs781868698	W269S	Decrease
rs782666368	Y103C	Decrease
rs1049717729	Y82N	Decrease
rs1336599647	V262A	Decrease
rs1453374014	S139N	Decrease
rs1487014301	W110G	Decrease
rs1556673736	I135N	Decrease
rs1556673810	W269C	Decrease

### 3.5. Conservatory Analysis of Shortlisted nsSNPs

Since nsSNPs can result in health problems for humans, knowledge of evolution is necessary to delve deeper into them [1]. Phylo3D was used to analyze the conservation profile of *AGTR2* amino acid residues in order to look into the possible effects of the 11 selected nsSNPs. It provided data for every residue in *AGTR2*, but we were more interested in the locations of the 11 nsSNPs that we found. As shown in Table 3, all the mutational residues were highly conserved and exposed except P271L, I135N, and V262A, which were predicted to be conserved based on Phylo3D prediction. These results suggested that the most harmful nsSNPs to the structure and function of the *AGTR2* protein are those found in highly conserved regions.

**Table 3:** Phylogenetic conservation profiling of respective nsSNPs.

SNP ID	Mutation	Prediction
rs3729979	P271L	Conserved
rs200599388	I132F	Highly Conserved
rs200855214	M138T	Highly Conserved
rs781868698	W269S	Highly Conserved
rs782666368	Y103C	Highly Conserved
rs1049717729	Y82N	Highly Conserved
rs1336599647	V262A	Conserved
rs1453374014	S139N	Highly Conserved
rs1487014301	W110G	Highly Conserved
rs1556673736	I135N	Conserved
rs1556673810	W269C	Highly Conserved

### 3.6. Selection of the Most Deleterious nsSNPs

The 11 most harmful nsSNPs were chosen, and the Robetta online tool was used to create 3D models of each mutant in order to predict alterations in the *AGTR2* protein. Each nsSNP substitution in the *AGTR2* protein sequence was carried out independently to create mutant protein 3D structures. After having 3D structures, RMSD and TM scores were calculated using TM‐Align for each of the mutant models. Although RMSD measurements display the average distance between *α*‐carbon backbones in wild and mutant models, the TM‐score quantifies topological similarity. Greater structural divergence between the mutant and wild type is indicated by higher RMSD values. With an RMSD value of 2.31 Å, the mutant I132F (rs200599388) had the highest value. W269C (rs1556673810), P271L (rs3729979), and I135N (rs1556673736) came in second and third, respectively. V262A (2.09 Å RMSD), Y103C (2.04 Å RMSD), M138T (1.97 Å RMSD), W269S (1.96 Å RMSD), Y82N (1.87 Å RMSD), S139N (1.42 Å RMSD), and W110G (1.31 Å RMSD) were among the other nsSNPs that showed smaller variation. Table 4 displays the RMSD values and TM scores.

**Table 4:** TM score and RMSD values of processed SNPs, predicted by TM‐align.

SNP ID	Mutation	TM‐score	RMSD
rs3729979	P271L	0.88500	2.23
rs200599388	I132F	0.90759	2.31
rs200855214	M138T	0.89682	1.97
rs781868698	W269S	0.90288	1.96
rs782666368	Y103C	0.88878	2.04
rs1049717729	Y82N	0.88527	1.87
rs1336599647	V262A	0.89714	2.09
rs1453374014	S139N	0.87533	1.42
rs1487014301	W110G	0.91415	1.31
rs1556673736	I135N	0.90759	2.18
rs1556673810	W269C	0.89380	2.29

After comprehensively shortlisting more likely deleterious nsSNPs from six different tools, encoded by different algorithms, we firstly shortlisted 18 nsSNPs. The structural and functional alterations due to these nsSNPs, their induced stability change on overall structure of *AGTR2* and conservation analysis, followed by topological similarity analysis (TM‐score), encouraged us to list the resulting four nsSNPs as the most deleterious among all the retrieved nsSNPs. Moreover, ClinVar database further evaluated all of the four shortlisted mutants as highly significant for clinical profiles, based on the already submitted information.

### 3.7. Secondary Structure Analysis of Proposed nsSNPs

The final selected four nsSNPs, along with the wild‐type *AGTR2*, were analyzed for secondary structure evaluation to understand more about their structural features. Using PSIPRED, we discovered that alpha‐helices make up the bulk of it, followed by beta strands and coils in all mutants and the wild‐type *AGTR2* (Table 5). This combination implies a separate structure with both hard and flexible components. Additional investigation using pCysMod revealed eight cysteine residues in all the query 2D structures, raising the possibility of disulfide connections that contribute to protein stability and stiffness.

**Table 5:** Showing secondary structure characteristics of respective sequences.

Candidate	*α*‐Helix (%)	*Β*‐strands (%)	Coils (%)
I132F	64.37	2.75	33.88
I135N	69.59	2.48	28.93
W269C	68.22	1.92	30.85
P271L	69.87	1.38	29.75
Wild type	68.32	2.48	29.20

### 3.8. Tertiary Structure Analysis

Four nsSNPs (I132F, W269C, P271L, and I135N) with the highest RMSD values were selected for remodeling, using AlphaFold. Using PyMOL, the four mutations are seen superimposed on top of the wild‐type *AGTR2* protein in Figure 4.

Figure 4(a) Superimposed mutant I132F structure along with wild_type *AGTR2*, (b) superimposed mutant I135N structure along with wild_type *AGTR2*, (c) superimposed mutant P271L structure along with wild_type *AGTR2*, (d) uperimposed mutant W269C structure along with wild_type *AGTR2*, and (e) structure of wild_type protein *AGTR2*.

**Figure:**
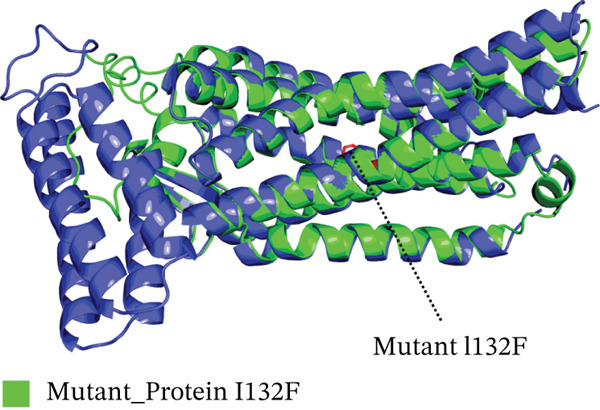
(a)

**Figure:**
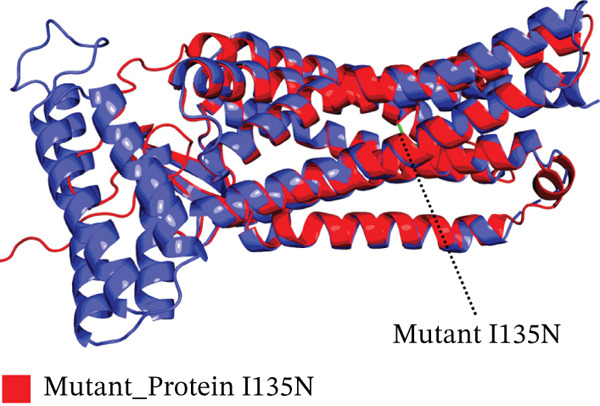
(b)

**Figure:**
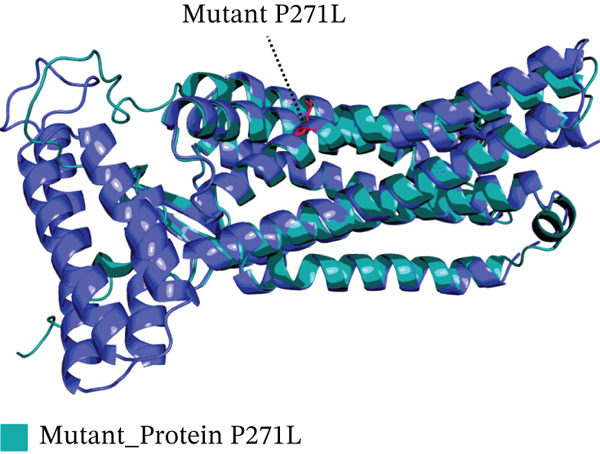
(c)

**Figure:**
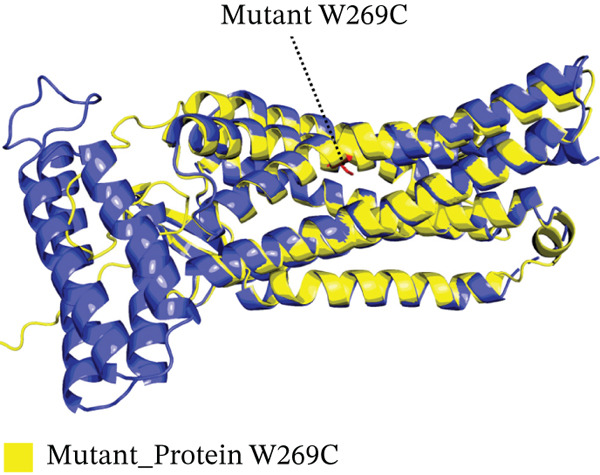
(d)

**Figure:**
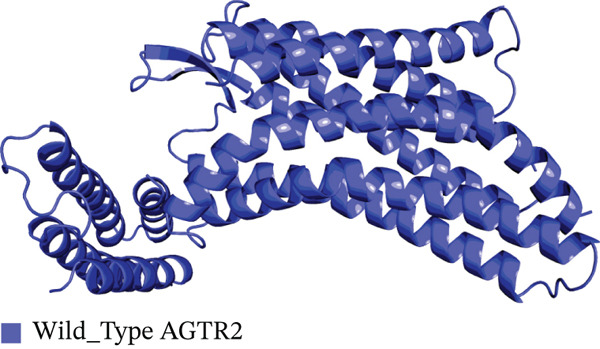
(e)

**Figure 5:**
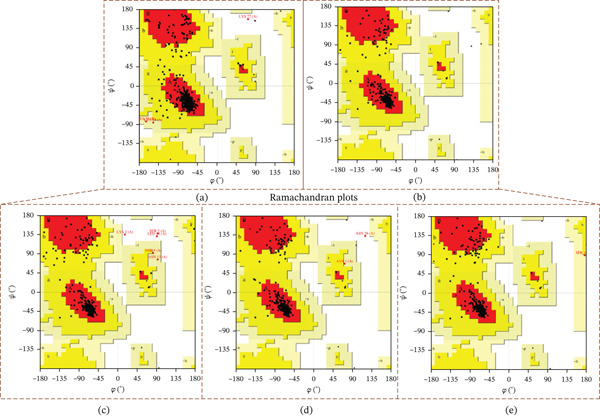
(a) Ramachandran plot of wild_type *AGTR2*. (b) Ramachandran plot of mutant I132F. (c) Ramachandran plot of I135N. (d) Ramachandran plot of P271L. (e) Ramachandran plot of W269C.

These 3D structures were then validated using Qmean, MolProbity, and the SAVES web‐based system. Qmean exhibited satisfactory results for all the predicted structures as the scores for I132F, I35N, W269C, and P271L were 0.75, 0.74, 0.75, and 0.76, respectively. Likewise, MolProbity provided reliable results for all the four mutated modeled proteins, along with their wild‐type *AGTR2*. For both the mutant and wild‐type *AGTR2* genes, the highest ERRAT scores were I132F = 95.20, I135N = 99.05, P271L = 98.11, and W269C = 98.43. Figure 5 shows the Psi and Psi degrees of the appropriate structures together with a Ramachandran plot of all mutations and *AGTR2*.

### 3.9. Molecular Docking

To assess the binding affinity and way of interaction between *AGTR2* mutants and ACE2 protein, we docked them together. We simulated this interaction using a computational program called ClusPro, developing 10 distinct models for each mutant–receptor combination, as well as the wild‐type *AGTR2*. One model from each complex was chosen. The wild‐type complex with ACE2 had the lowest energy (−1479.2) and the greatest number of cluster members (71), indicating a stable and favorable binding. Following that, the W269C‐ACE2 complex had a binding energy of −1341.5 and 53 cluster members, whereas the I132F‐ACE2 complex had a lower binding affinity (−1238.6) and 59 cluster members. Finally, mutants P271L and I135N had the lowest binding affinities (−1173.0 and −1152.6, respectively) and cluster membership (33 and 47, respectively). These results (Table 6) indicated that all the mutants had the lowest binding affinity with ACE2 (as compared with the wild‐type *AGTR2*) and the greatest potential to disrupt their binding mechanism, resulting in cardiovascular diseases.

**Table 6:** The binding energies along with the cluster members of all complexes.

Mutant	Binding energy	Cluster members
I132F	−1238.6	59
I135N	−1115.6	51
W269C	−1341.5	33
P271L	−1173.0	53
Wild type	−1479.2	71

Moreover, Haddock was also utilized to redock all the mutants, along with wild type, with the ACE2 receptor. This was applied to validate the previous docking results of ClusPro. According to the haddock evaluation, the best cluster out of total 10 from each of the docked complexes was selected based on its lowest energy (i.e., the highest binding affinity). Surprisingly, from all the mutants, I132F exhibited the lowest binding affinity with ACE2 (i.e., −98.2 ± 8), indicating their weaker interactions (Table 7). Following this, I135N, W269C and P271L also demonstrated weaker interactions with ACE2 receptor (i.e., −99.8 ± 3.7, −102.3 ± 4.3, −104.8 ± 5.1). Meanwhile, comparably higher binding affinity (−123.5 ± 6.4) of wild‐type *AGTR2* with ACE2 receptor suggested that all the mutants have weaker binding mechanism with ACE2, which may disrupt to induce cardiovascular diseases.

**Table 7:** The binding energies along with the cluster members of all complexes.

Mutant	Binding energy
I132F	−98.2 ± 8
I135N	−99.8 ± 3.7
W269C	−102.3 ± 4.3
P271L	−104.8 ± 5.1
Wild type	−123.5 ± 6.4

Further analysis with PDBsum showed a network of connections between the complexes. We evaluated results based on three interactions: nonbonded contacts, which contribute to overall attraction; salt bridges, which are specific interactions involving the charged atoms that strengthen the binding; and hydrogen bonds, which form precise connections between molecules, similar to tiny bridges. The I135N mutant exhibited the fewest interactions with the ACE2 receptor, with just six hydrogen bonds, 142 nonbonded contacts, and two salt bridges. Following this, the I132F mutant had the fewest interactions (relative with the wild type), establishing 11 hydrogen bonds, 120 nonbonded contacts, and three salt bridges. Furthermore, P271L and W269 displayed fewer interactions with the ACE2 receptor, such as hydrogen bonds (three), nonbonded contacts (211 and 219, respectively), and salt bridges (three). Finally, wild‐type *AGTR2* complexed with ACE2 produced the most interactions, creating 15 hydrogen bonds, 261 nonbonded contacts, and three salt bridges (Figure 6). These findings supported the poorer interactions of *AGTR2* mutants with ACE2 relative to the wild type. As a result of the loss of greater binding affinities, these weaker connections may be a more likely cause of cardiovascular illness.

Figure 6(a) Wild_type *AGTR2*‐ACE2 complex, along with their mutual interactions. (b) Mutant I132F‐ACE2 complex, along with their mutual interactions. (c) Mutant I135N‐ACE2 complex, along with their mutual interactions. (d) Mutant W269C‐ACE2 complex, along with their mutual interactions. (e) Mutant P271L‐ACE2 complex, along with their mutual interactions.

**Figure:**
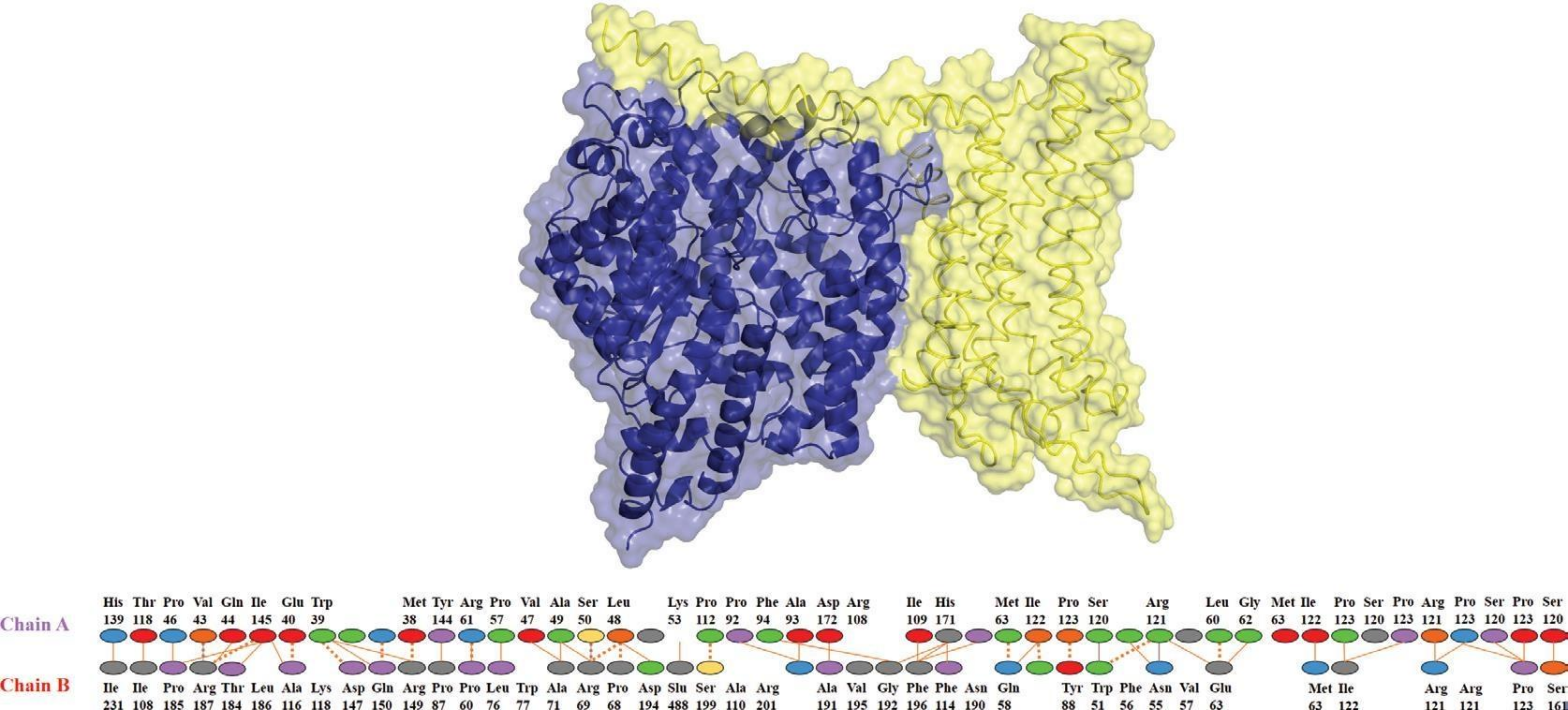
(a)

**Figure:**
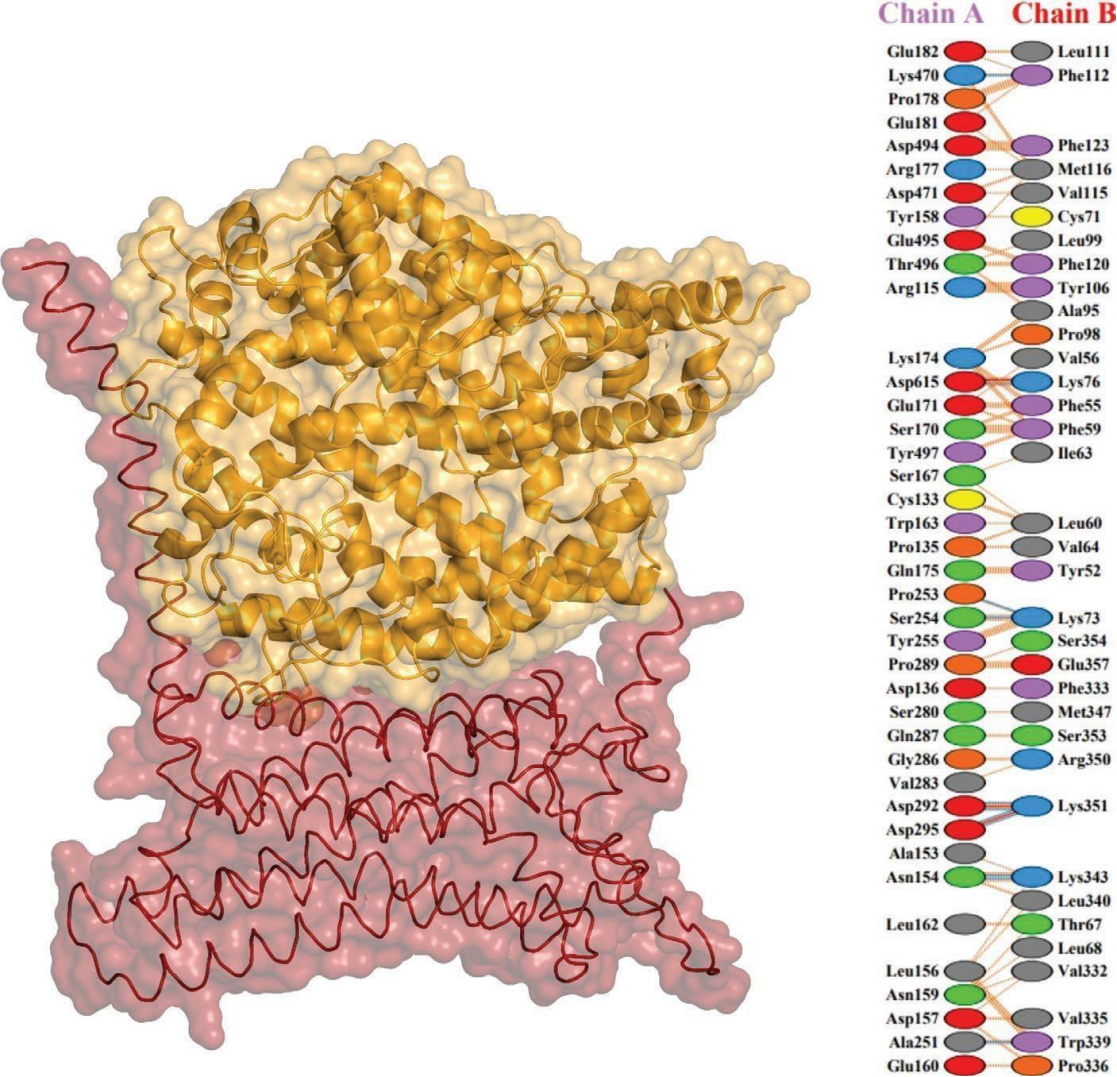
(b)

**Figure:**
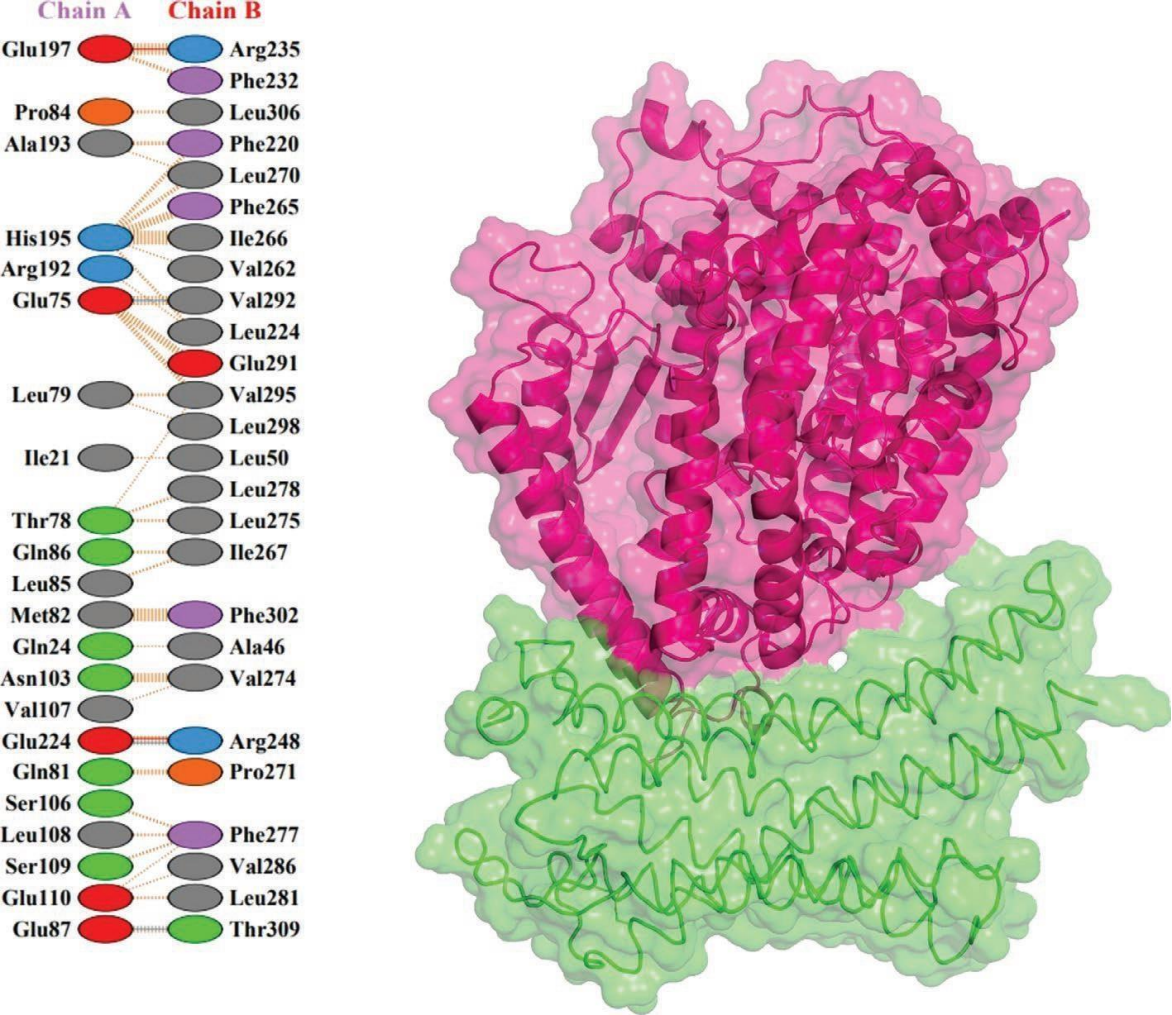
(c)

**Figure:**
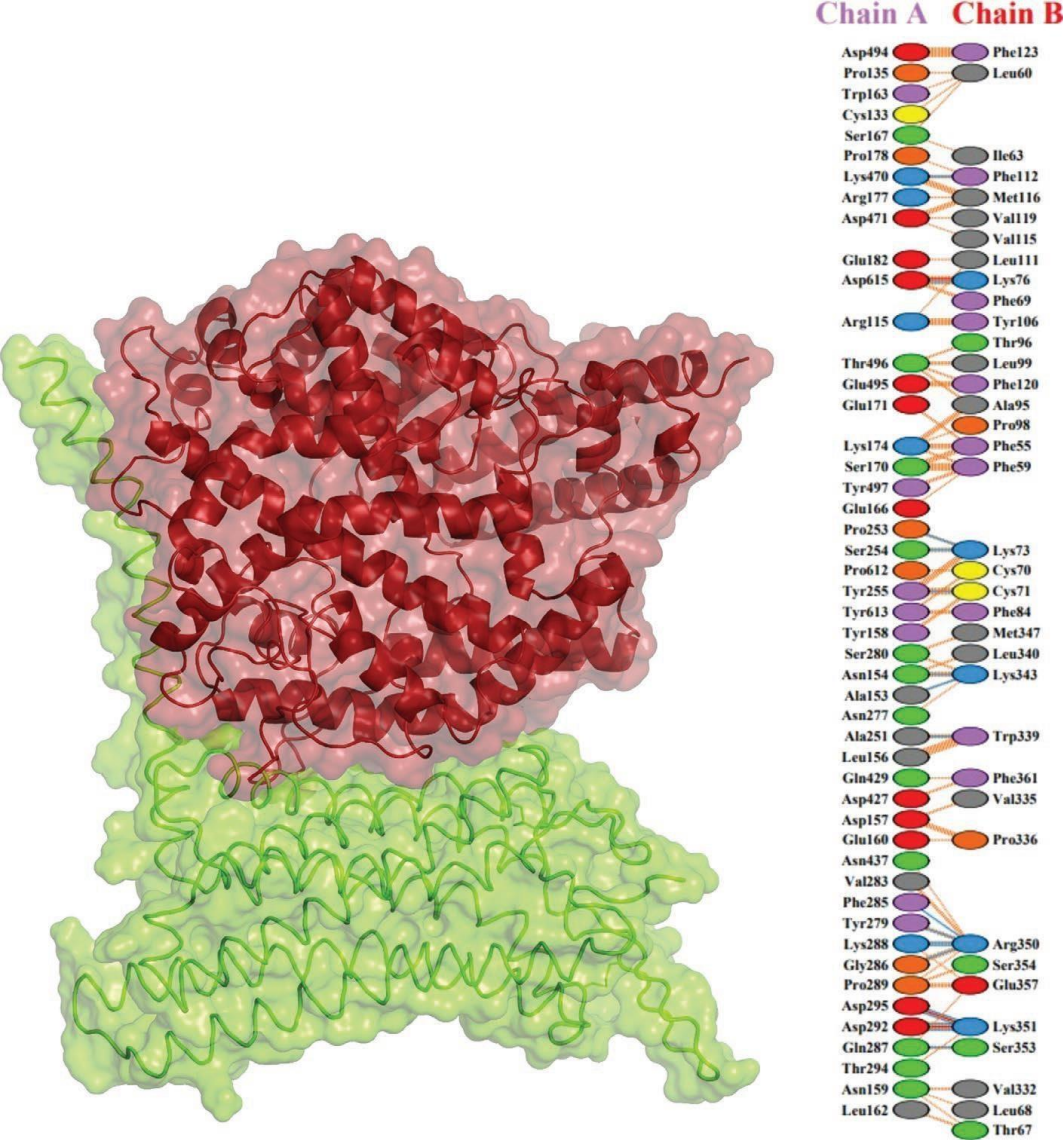
(d)

**Figure:**
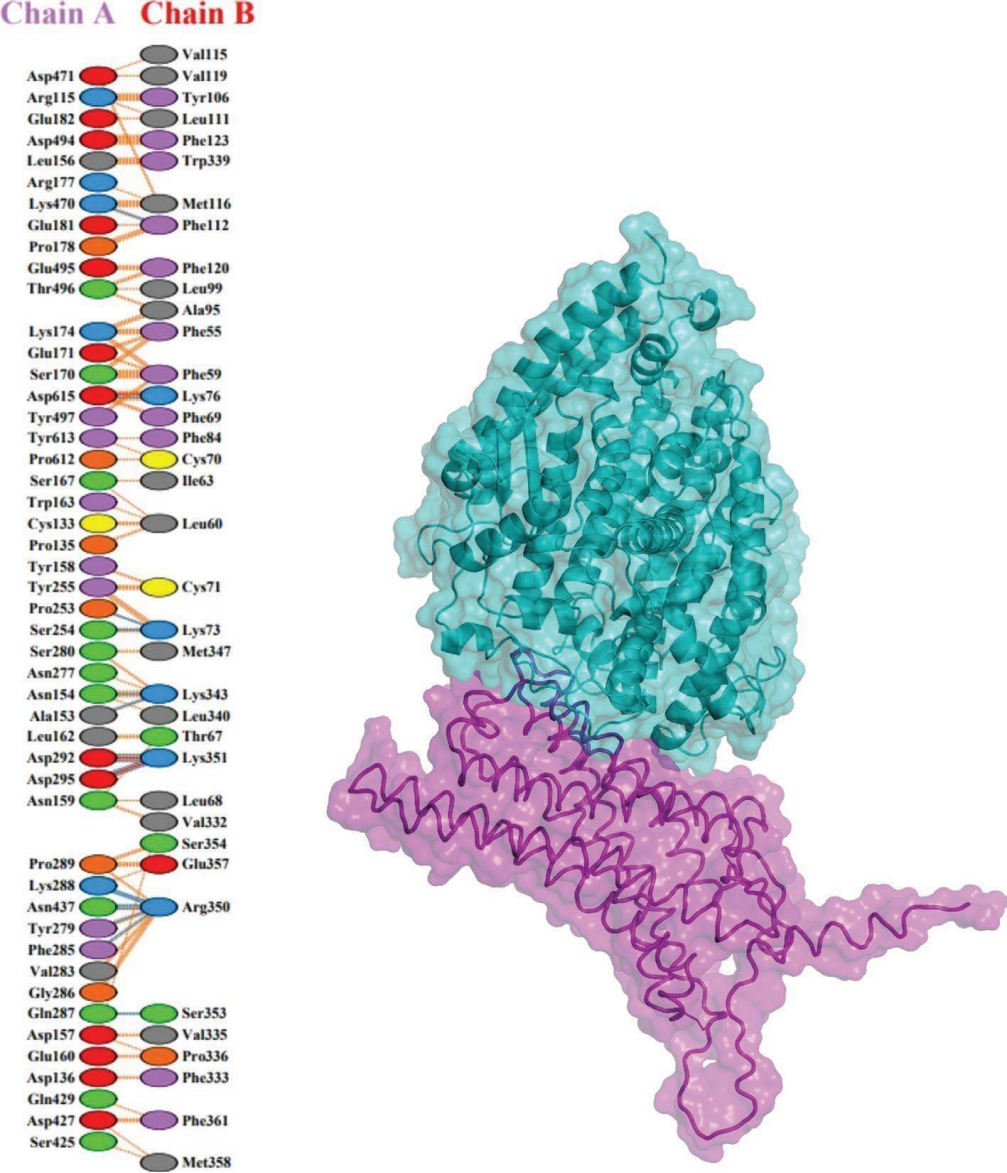
(e)

### 3.10. MD Simulation

In contrast to the wild type, the I132F, I135N, W269C, and P271L mutants accomplished less stable ACE2 complexes (PDB ID: IR42). MD simulation using the AMBER package was employed to explore the time‐dependent change of bound protein–protein complexes up to 200 ns. The degree of degenerative changes in protein–protein complex and dynamic behavior was assessed using particular measures such as RMSD, root mean square fluctuation (RMSF), and radius of gyration (Rg). Using receptor ACE2, four mutants and their wild type were examined using MD simulation to estimate their RMSD, RMSF, *β*‐factor, principal component analysis (PCA), free energy landscape (FEL) calculations, and Rg to explore the structural dynamics of protein–protein complexes up to 200 ns.

#### 3.10.1. RMSD Analysis

To maximize proteins′ therapeutic potential, binding stability must be established using molecular simulations. It also impacts the degree to which proteins interact with one another. Furthermore, binding stability is necessary for molecular optimization of novel proteins so that prospective targets may be correctly assessed. The binding stability was evaluated by assessing the simulated trajectories and calculating the RMSD as a function of time [62]. We assessed and tested the stability of each optimized hit in this case. A study of the four chosen mutant complexes shows that they all have extremely unstable behavior with the ACE2 receptor as compared with the natural type. The wild‐type complex with ACE2 had the best stability relative to other mutants, with an average RMSD of 2–4 Å over 200 ns (Figure 7a). For the first 60 ns, the RMSD was constant at 2 Å, with a simultaneous rise up to 4 Å between 60 and 90 ns. The simulated trajectory showed very stable RMSD values (~4 Å) for the rest of the simulation, suggesting a highly stable combination between wild‐type *AGTR2* and the ACE2 receptor. In contrast, the RMSD of the P271L‐ACE2 complex fluctuated throughout 200 ns. The RMSD increased up to 7.8 Å at 40 ns, then fell abruptly between 40 and 60 ns (Figure 7b). It continued to deviate for the rest of the simulation, indicating a very unstable P271L‐ACE2 complex as compared with the wild type. Similarly, the W269C‐ACE2 complex showed some variation, increasing up to 5.5 Å until 50 ns, followed by a gradual fall to 4 Å till 160 ns. After then, it displayed modest deviations until 200 ns, showing that the W269C‐ACE2 complex was less stable than the wild type (Figure 7c). Finally, I135N‐ACE2 and I132F‐ACE2 complexes exhibited less divergence than other mutants (~4 Å). SNPs disrupt the interaction between *AGTR2* and ACE2 receptors, resulting in less stable complexes compared with the wild type (Figure 7d,e). Mutations may disturb biological processes, which might lead to a variety of cardiovascular disorders.

Figure 7(a) Representing RMSD of wild‐type ACE2 complex, (b) representing RMSD of P271L‐ACE2 complex, (c) representing RMSD of W269C‐ACE2 complex, (d) representing RMSD of I135N‐ACE2 complex, and (e) representing RMSD of I132F‐ACE2 complex.

**Figure:**
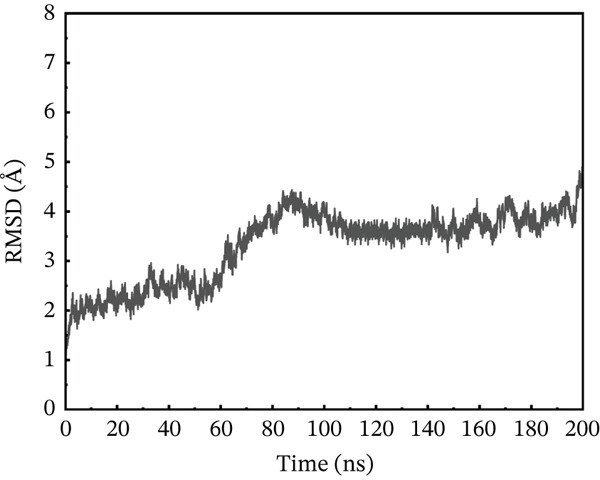
(a)

**Figure:**
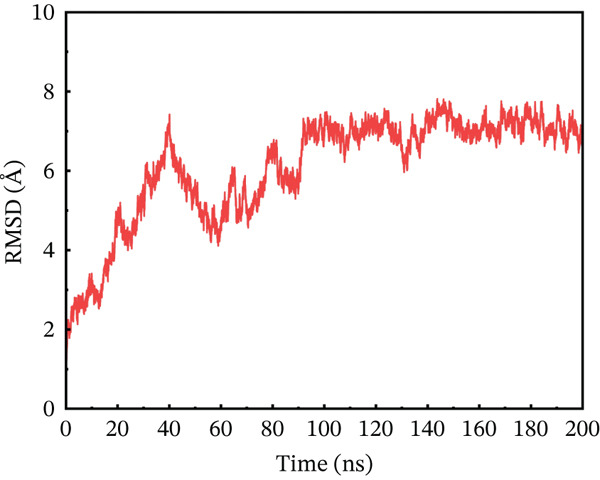
(b)

**Figure:**
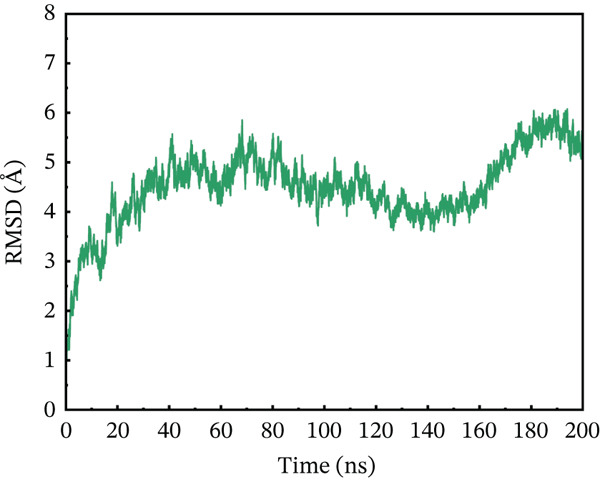
(c)

**Figure:**
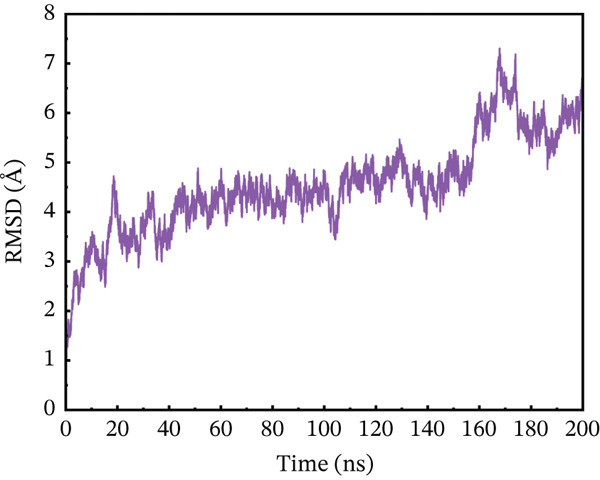
(d)

**Figure:**
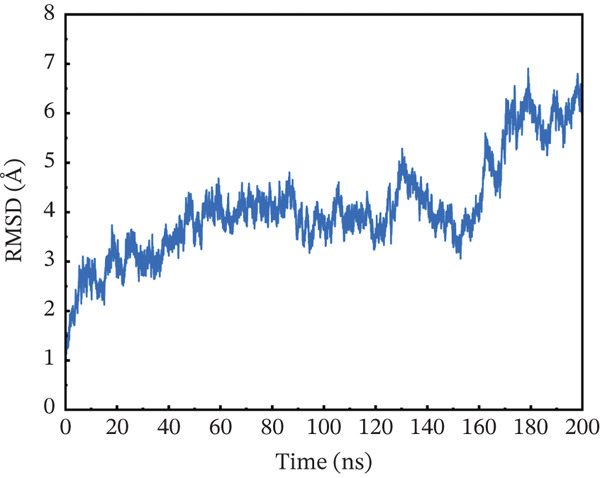
(e)

#### 3.10.2. RMSF Analysis

RMSF analysis was performed on individual amino acids to have a better understanding of the stability of ACE2 active site residues during protein interaction [63]. This analysis was carried out across the whole 200 ns MDs trajectory, as shown in Figure 8. All mutants, including the wild type, had their RMSF values calculated. The average RMSF of each system differed from one another. The wild‐type compound with the ACE2 receptor had the lowest RMSF values, remaining around 3.5 Å throughout the simulation. However, several residues between 600 and 680 showed RMSF around 6 Å. The least fluctuations revealed a fairly stable compound between wild‐type *AGTR2* and the ACE2 receptor (Figure 8a). On the other hand, the P271L‐ACE2 complex exhibited major variations, indicating instability. The first 600 residues were maintained quite typical. The remaining residues had the largest fluctuation values, including a destructive fluctuation of up to 22 Å, suggesting instability relative to the wild type (Figure 8b). Following this, the W269C‐ACE2 complex demonstrated comparable stability with the wild type. The residues after 600 indexes exhibited significant changes, with the largest fluctuation reaching 18 Å, indicating instability (Figure 8c). Similarly, I135N‐ACE2 and I132F‐ACE2 complexes showed minor variations. The average RMSF ranged from 2 to 4.5 Å, with certain residues (600–680) fluctuating up to 12 Å in both complexes (Figure 8d,e). The total RMSF of all the complexes showed that mutant complexes were less stable than wild‐type complexes, indicating their function in disturbing the normal RAS mechanism and perhaps contributing to cardiovascular disease. Further insights through Rg, PCA, and FEL analyses were suggested to validate this behavior.

Figure 8(a) Representing RMSF of wild‐type ACE2 complex ,(b) representing RMSF of P271L‐ACE2 complex, (c) representing root mean square fluctuation of W269C‐ACE2 complex, (d) representing RMSF of I135N‐ACE2 complex, and (e) representing RMSF of I132F‐ACE2 complex.

**Figure:**
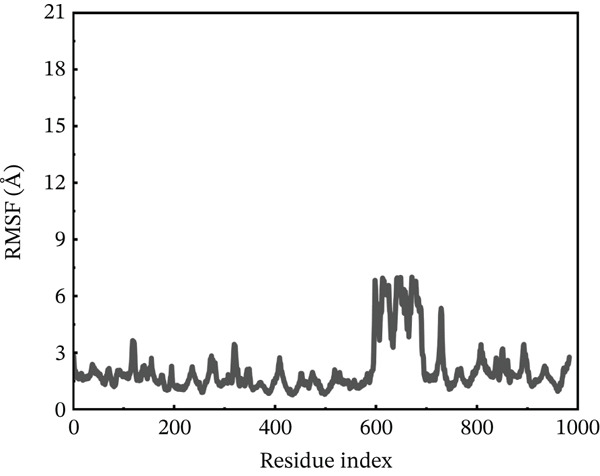
(a)

**Figure:**
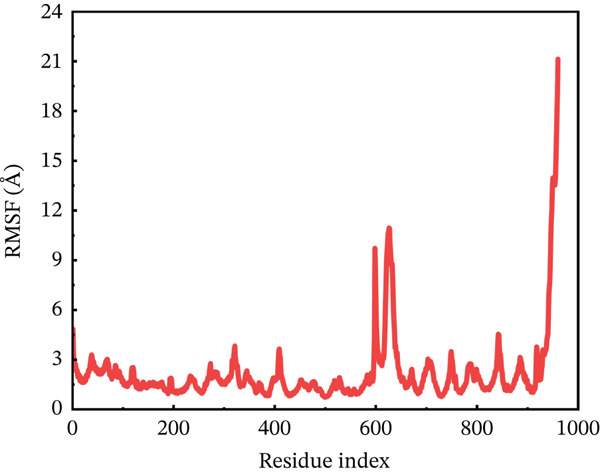
(b)

**Figure:**
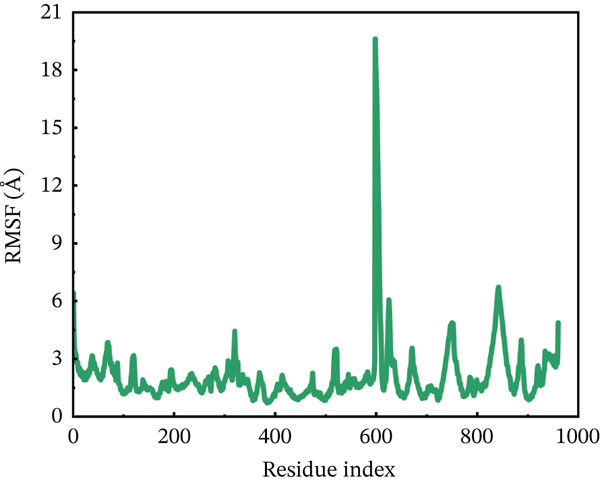
(c)

**Figure:**
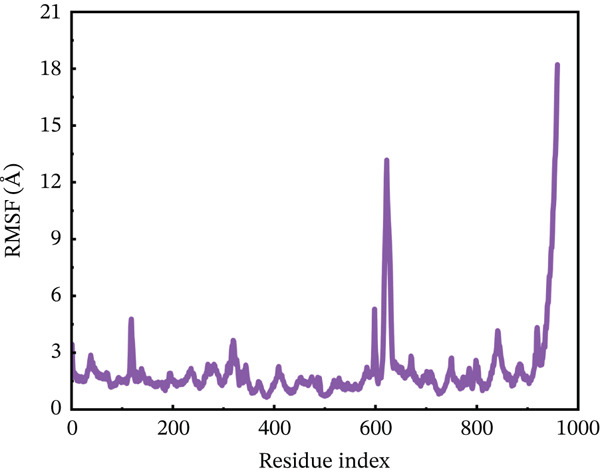
(d)

**Figure:**
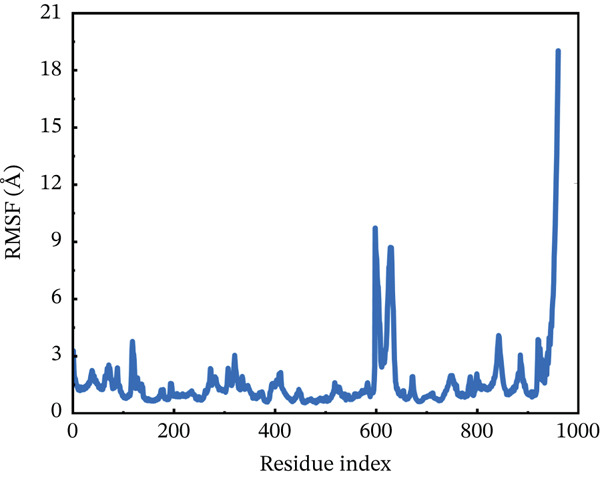
(e)

#### 3.10.3. Rg Analysis

We assessed the structural compactness of each complex in a dynamic scenario to better understand its dynamics and the binding and unbinding processes that occurred throughout the simulation. To do this, we estimated the Rg as a function of time [64]. The Rg values for the wild type and ACE2 complex showed the highest compactness, interacting at 48.2 Å throughout the simulation (Figure 9a). In contrast, the P271L‐ACE2 complex was a less stable conformation, with values ranging from 63.4 to 63.55 Å, indicating less interaction between the mutant and receptor (Figure 9b). Similarly, W269C‐ACE2 had a low Rg range of 52.2–52.4 Å, indicating weaker binding compared with wild type (Figure 9c). Furthermore, I135N exhibited reduced compactness between Rg values at 51.5 and 51.65 Å (Figure 9d). The I132F‐ACE2 complex performed well but had lower compactness than the wild type, ranging from 48.3 to 48.45 Å (Figure 9e). Overall, the data revealed that the natural type had the maximum compactness compared with mutants. This indicated that the mutations interact with the ACE2 receptor with a comparable lower binding affinity.

Figure 9(a) representing Rg of wild‐type ACE2 complex, (b) representing Rg of P271L‐ACE2 complex, (c) representing Rg of W269C‐ACE2 complex, (d) representing Rg of I135N‐ACE2 complex, and (e) representing Rg of I132F‐ACE2 complex.

**Figure:**
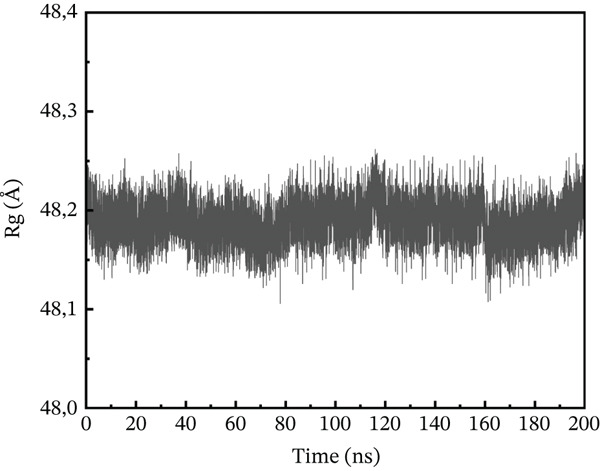
(a)

**Figure:**
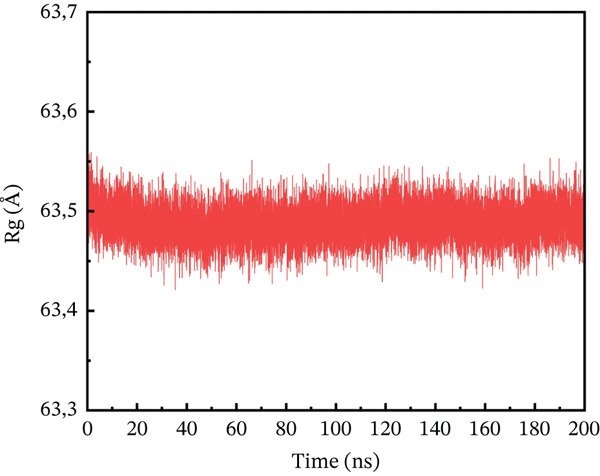
(b)

**Figure:**
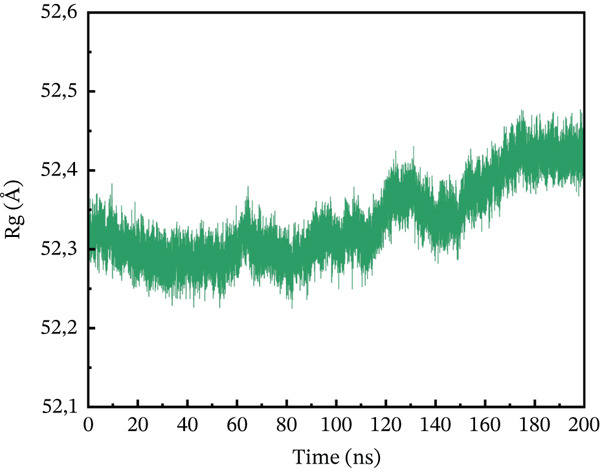
(c)

**Figure:**
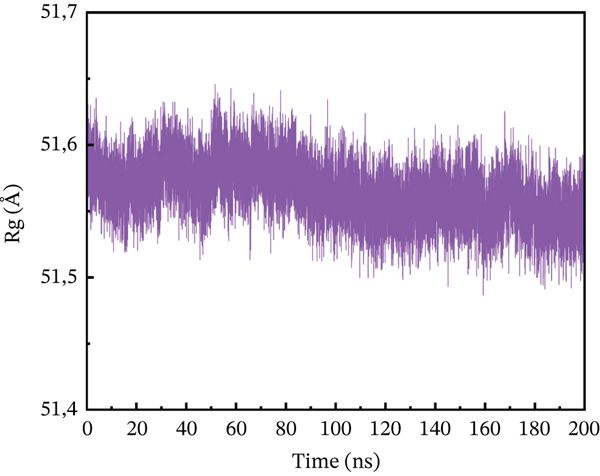
(d)

**Figure:**
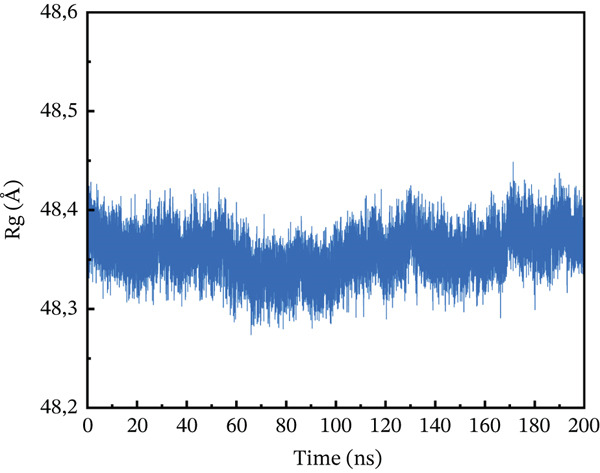
(e)

#### 3.10.4. *β*‐Factor Analysis

The *β*‐factor is a thermal disorderliness calibration function used in RMSF to measure atomic position‐specific structural stability. Protein thermal stability and overall fluctuation are caused by the most oscillations and vibrations of all atoms [65]. Higher *β*‐factor values indicate lower structural stability. The wild‐type ACE2 complex exhibited the most stable *β*‐factor values, ranging from 25 to 35 nm, over nearly all residues. Only residues between 600 and 680 exhibited considerable variation in overall structure (Figure 10a). The P271L‐ACE2 complex had variable beta‐factor values, with certain residues reaching 1200 nm. This indicates poor protein–protein interaction (Figure 10b). The W269C‐ACE2 complex likewise showed unstable beta‐factor values, with certain residues reaching up to 1000 nm (Figure 10c). The I135N‐ACE2 and I132F‐ACE2 complexes behaved similarly to W269C‐ACE2, with beta‐factor values increasing to 950 nm (Figure 10d,e), indicating considerable instability relative to the wild type. Overall, beta‐factor data showed that the wild‐type ACE2 complex was the least disordered of all the mutant complexes.

Figure 10(a) Representing *β*‐factor of wild‐type ACE2 complex, (b) representing *β*‐factor of P271L‐ACE2 complex, (c) representing *β*‐factor of W269C‐ACE2 complex, (d) representing *β*‐factor of I135N‐ACE2 complex, and (e) Representing *β*‐factor of I132F‐ACE2 complex.

**Figure:**
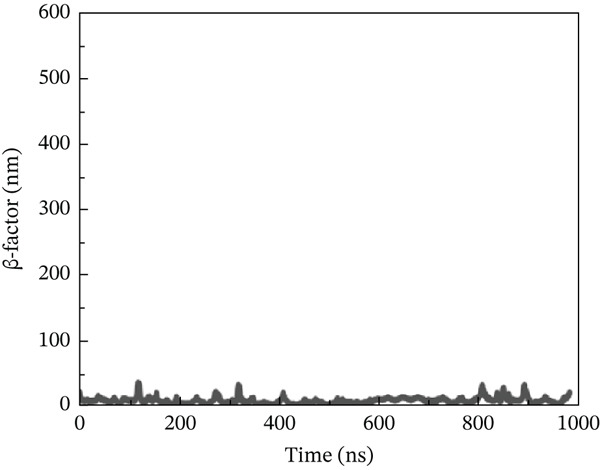
(a)

**Figure:**
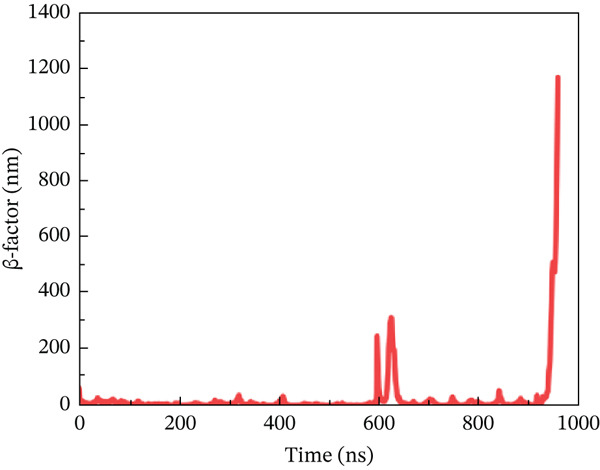
(b)

**Figure:**
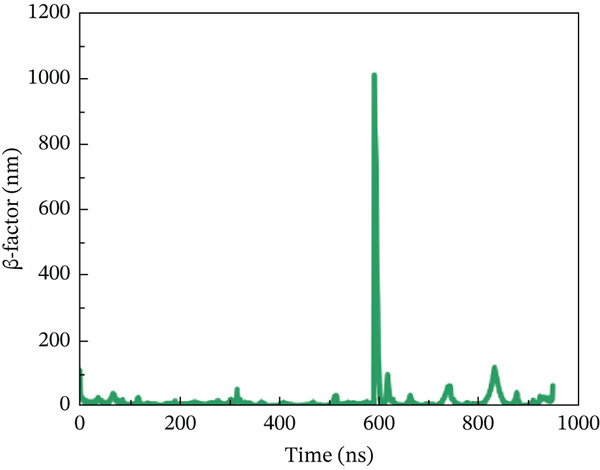
(c)

**Figure:**
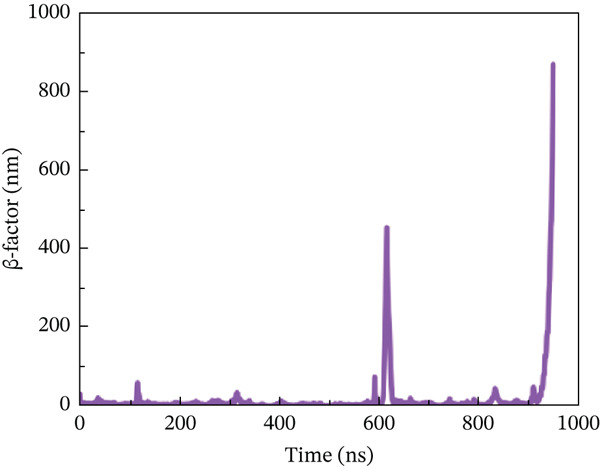
(d)

**Figure:**
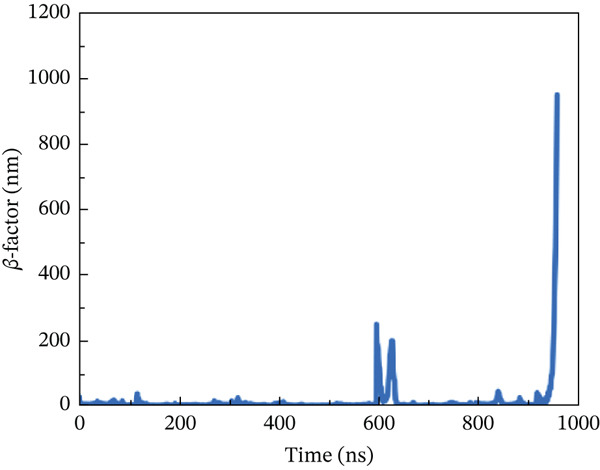
(e)

#### 3.10.5. PCA

PCA was performed on 200 ns MD trajectories to evaluate the conformational changes associated with mutant–receptor interactions. All complexes exhibited distinct conformational dynamics while maintaining common cluster‐like motion patterns (Figure 11 and Supporting Material S1). The wild‐type ACE2 complex showed a broader range of motion, with PC1 values spanning from −300 to +400 and PC2 values from −200 to +200, indicating greater conformational flexibility. In contrast, the mutant‐ACE2 complexes displayed more restricted motions on average, with PC1 values between −300 and +300 and PC2 values between −100 and +200. These observations suggest that wild‐type binding to ACE2 promotes more extensive conformational sampling, whereas *Δ*mutants‐ACE2 interactions limit the dynamic range, potentially stabilizing specific conformational states. The observed differences in the principal component distributions highlight the impact of mutations on protein–protein dynamics and their influence on complex stability.

Figure 11(a) PCA profile of wild‐type *AGTR2*, complexed with ACE2, (b) PCA profile of mutant P271L‐*AGTR2*, complexed with ACE2, (c) PCA profile of mutant W169C‐*AGTR2*, complexed with ACE2, (d) PCA profile of mutant I135N‐*AGTR2*, complexed with ACE2, and (e) PCA profile of mutant I132F‐*AGTR2*, complexed with ACE2.

**Figure:**
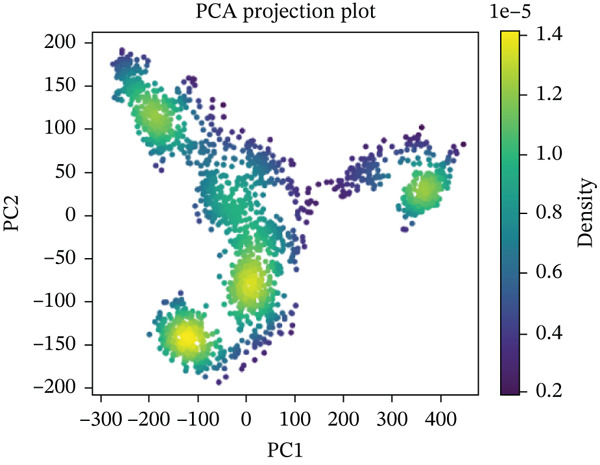
(a)

**Figure:**
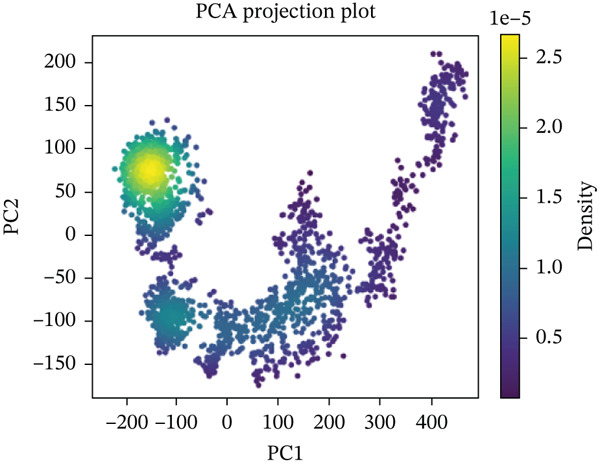
(b)

**Figure:**
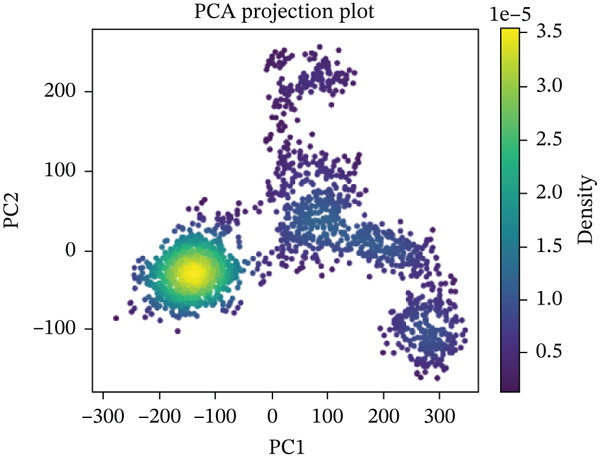
(c)

**Figure:**
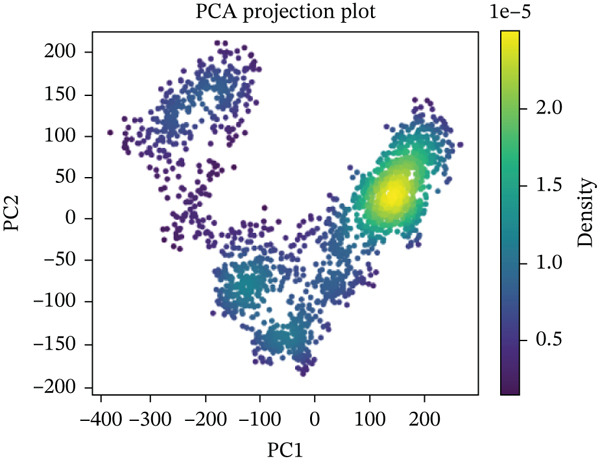
(d)

**Figure:**
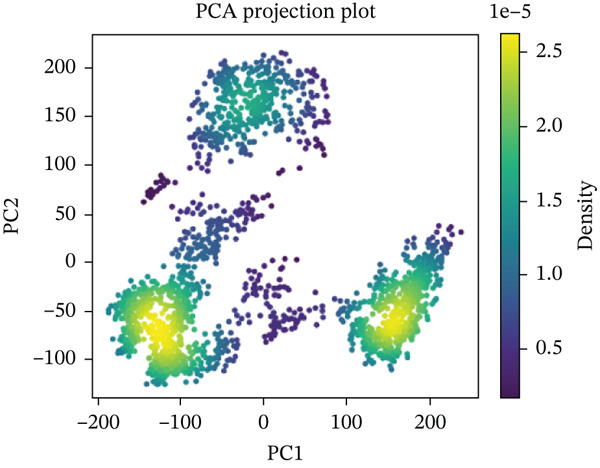
(e)

#### 3.10.6. FEL Analysis

**Figure 12:**
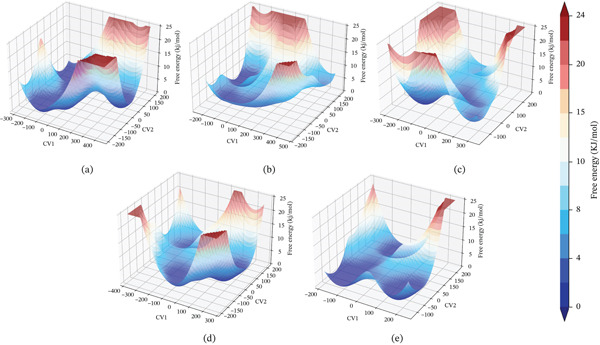
(a) FEL profile of wild‐type *AGTR2*, complexed with ACE2, (b) FEL profile of mutant P271L‐*AGTR2*, complexed with ACE2, (c) FEL profile of mutant W169C‐*AGTR2*, complexed with ACE2, (d) FEL profile of mutant I135N‐*AGTR2*, complexed with ACE2, and (e) FEL profile of mutant I132F‐*AGTR2*, complexed with ACE2.

FEL analysis provides a critical framework for evaluating the conformational stability and dynamic behavior of protein complexes during MD simulations. By mapping the free energy distribution as a function of collective variables, FEL reveals the thermodynamically favorable states and transition pathways that characterize the conformational ensemble of the system. In this study, the FELs of the wild type and mutant *AGTR2*‐ACE2 complexes were constructed to assess their energetic stability and structural adaptability (Figure 12 and Supporting Material S1). The color gradient, ranging from deep blue (indicating low free energy and high stability) to red (high free energy and reduced stability), delineates the energy profile of each system. The wild‐type *AGTR2*‐ACE2 complex exhibited a well‐defined energy basin with deep minima, indicative of a stable conformational state and stronger interaction stability. In contrast, the FELs of all three mutant complexes revealed more rugged topographies, characterized by multiple shallow and dispersed energy wells. This pattern reflects increased conformational heterogeneity and elevated energy fluctuations, suggesting a reduction in structural stability relative to the wild‐type complex. These findings imply that the deleterious mutations compromise the thermodynamic stability of *AGTR2* in complex with ACE2, potentially weakening their binding affinity and perturbing functional interactions. Such destabilization may contribute to the pathophysiological mechanisms underlying cardiovascular dysfunctions associated with these mutations.

### 3.11. Predicted PTMs

#### 3.11.1. Methylation

GPS‐MSP 3.0 was used for this purpose, which predicted no sites in *AGTR2* to be methylated.

#### 3.11.2. Phosphorylation

As seen in Figure 13, *AGTR2* phosphorylation sites were predicted using GPS 3.0 and NetPhos 3.1. Fifty‐one residues (Ser:20, Thr:19, TyrL:12) were predicted by NetPhos 3.1 to be potentially phosphorylated. Nevertheless, 13 residues (Ser:07, Thr:05, Tyr:00) were identified by GPS 3.0 as possibly phosphorylated.

Figure 13(a) Phosphorylation graph of *AGTR2* residues and (b) ubiquitination graph for *AGTR2* residues.

**Figure:**
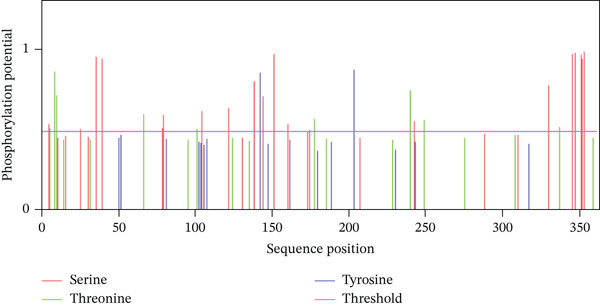
(a)

**Figure:**
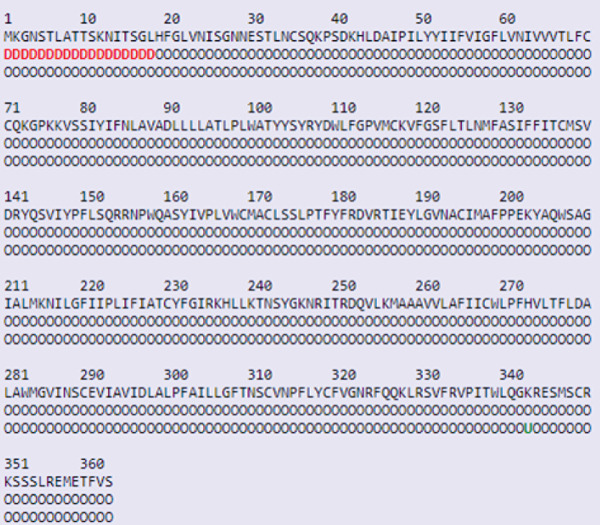
(b)

#### 3.11.3. Ubiquitylation

The ubiquity forecast was done using the RUBI web platform. Out of a total of 17, it predicted that one lysine residue would be ubiquitinated. The predicted residue was not found in a deleterious or highly conserved nsSNP region. It is estimated that 5.882% of protein is ubiquitinated overall.

#### 3.11.4. Glycosylation

The most likely glycosylation sites were estimated using NetOGlyc4.0. The wild‐type *AGTR2* protein was found to have glycosylated positions at Positions 25, 6, 9, 10, 11, 15, 16, and 32, with scores of 0.72, 0.58, 0.73, 0.64, 0.50, 0.62, 0.53, and 0.54, respectively. It is anticipated that these sites will be glycosylated.

### 3.12. Allelic Frequency of Proposed Mutants

To determine the allelic frequency of suggested mutants, we employed gnomAD (genome aggregation database), which is a large‐scale database providing data of different populations that can help us understand the prevalence of proposed variants in different regions. According to the gnomAD database, P271L is the most frequent allele found in almost 26 genomes and 411 exomes, followed by 132F, which has been reported in 124 genomes and 139 exomes. On the other hand, I135N and W269C were reported to be found in only one genome in the European region. The relevant information about allelic frequency is written in Table 8.

**Table 8:** Representing the allelic frequency of suggested nsSNPs.

Mutant	Genomes	Exomes	Total
I132F	124	139	263
I135N	1	0	1
W269C	1	0	1
P271L	26	411	437

### 3.13. Gene–Gene Interaction

The interactions between the *AGTR2* gene and other genes were predicted using GeneMANIA and STRING. *AGTR2* has physical interactions with HTR2B, AGT, EDN1, REN, COL1A1, AGTR1, TIMP3, ACE, AGTRAP, MTUS1, RXFP, PTPN6, and PIK3CB, according to the GeneMANIA data. AGER, REN, EDN1, COL1A1, HTR2B, TIMP3, AGTR1, ACE, PTPN6, IRF1, WT1, HOXA4, CLCNKB, and SFTPB are among the genes with which *AGTR2* is expressed in common. IRF1, WT1, HOXA4, ELN, CLCNKB, SFTPB, AGER, REN, ACE, EDN1, TIMP3, and COL1A1 are colocalized with it. It communicates via pathways with AGT, PTPN6, PIK3CB, AGTR1, EDN1, and IRF1. TIMP3, AGT, MTUS1, AGER, ELN, REN, AGTRAP, CLCNKB, IRF1, EDN1, AGTR1, RXFP, and SFTPB are among the genes that interact. Additionally, it has protein domains in common with RXFP4, AGTR1, and HTR2B. STRING predictions showed a cumulative score for every gene. Figure 14a,b displays the gene interactions predicted by GeneMANIA and STRING, respectively.

Figure 14(a) Gene–gene interaction of *AGTR2* with other closely linked genes, predicted by GeneMania and (b) gene–gene interaction of *AGTR2* with other closely linked genes, predicted by STRING.

**Figure:**
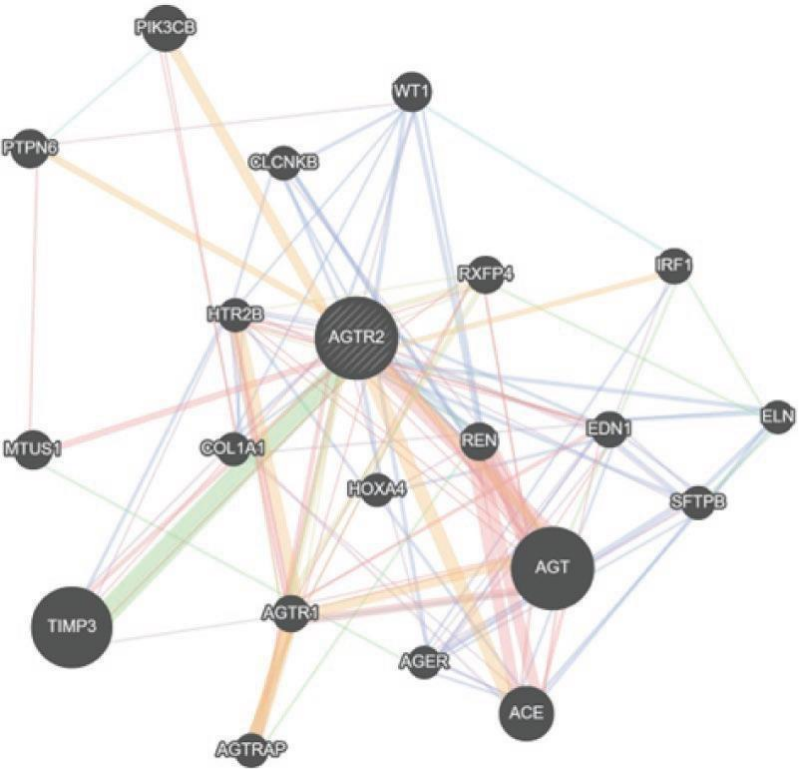
(a)

**Figure:**
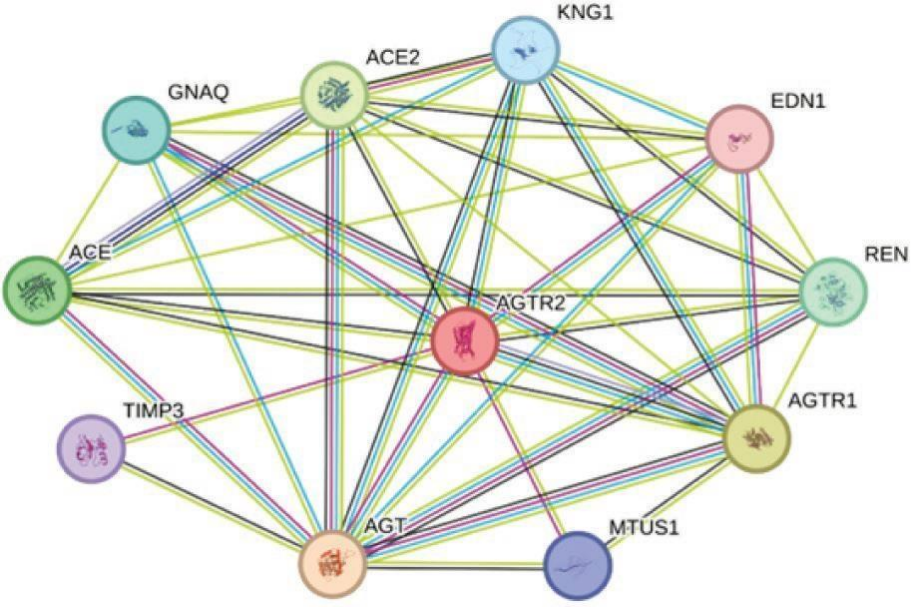
(b)

## 4. Discussion

Numerous studies have connected nsSNPs in *AGTR2* to different cardiovascular disorders [11, 12]. Presumably, a large number of nsSNPs do not affect function and are neutral. Due to their effects on protein functional regions or interference with protein folding, several of these nsSNPs are probably harmful [66]. Understanding the importance of *AGTR2* gene SNPs and their correlation with diseases is therefore essential. The current study employed in silico analysis to determine the effect of potentially harmful nsSNPs on the *AGTR2* protein. This study investigated the SNPs of the *AGTR2* gene because these variants may have significance in cardiovascular diseases such as coronary artery disease. Just 20.81% of the *AGTR2* gene′s SNPs are nsSNPs, 3% are 5 ^′^UTR SNPs, and 32% are 3 ^′^UTRs. No additional SNPs from other SNP categories were included in our analysis. Consequently, the number of SNPs that have a direct impact on the *AGTR2* protein is quite low. The 18 nsSNPs that were predicted to have a negative impact were shown to be the most harmful nsSNPs across all detection approaches. Out of the 104 nsSNPs predicted by SIFT, Polyphene‐2 (81 nsSNPS) was the most harmful. The Top 19 most harmful nsSNPs were predicted by CADD. Twenty‐three nsSNPs were shown by MetaLR to be harmful. In this investigation, the most damaging nsSNPs selected are cross‐checked using Ensemble genome browser tools like Mutation Assessors and REVEL. Revel claimed that 44 nsSNPs might be dangerous; however, Mutation Assessor identified 28 SNPs as potentially troublesome. Many features, including acetylation gain, methylation losses, changed interfaces, and intrinsic disorder gain, are predicted by MutPred1.2. With *p* values of 0.937, 0.928, and 0.918, respectively, the SNPs W110G, W269C, and W269S were the highest; Y103C, P271L, and Y82N came in second and third, with 0.873, 0.861, and 0.837, respectively. SNPs R251H and R251C exhibited the lowest *p* values (0.316 and 0.396, respectively), according to the data. The results suggest that these nsSNPs might affect the *AGTR2* protein. I‐Mutant was used to investigate how nsSNPs affect the stability of proteins. The RI values, which represent the lowest to the highest reliabilities, are presented for the outcomes and range from 0 to 10. Every nsSNP that was assessed using the I‐Mutant technique was found to reduce protein stability, as shown in Table 2. Phylo3D states that highly conserved amino acids are essential for the structure and function of proteins. The longer an amino acid is conserved in a protein, the more important it is thought to be in terms of interaction. According to a study, conservation zones contain the most dangerous nsSNPs [67]. The P271L, V262A, and I135N mutations are conserved, but the other mutations are not, according to the Phylo3D tool′s analysis. The altered *AGTR2* protein structures were simulated using Robetta, and the FASTA protein sequences were provided. Four models were produced for each mutational sequence. Afterwards, we assessed these 3D models using Qmean, MolProbity, and Saves Server. Next, the TM‐align algorithm looked at each nsSNP best models and used its method to calculate each structure′s RMSD value. The five selected nsSNPs had large RMSDs, indicating that they were the most harmful SNPs in the *AGTR2* gene. Significant differences between mutant and wild‐type proteins are indicated by RMSD values greater than 2 Å. PTMs are important regulators of protein function, which includes PPIs and cell signaling [68]. We also employed molecular docking techniques to validate the shortlisted nsSNPs role in cardiovascular diseases, by docking them with ACE2 receptor. The ACE2 receptor is the most important protein, which maintains the RAS signaling in *AGTR2*. We proposed that disruption in the interactions between *AGTR2* and ACE2 may be a cause of cardiovascular diseases. After docking with ACE2, we further validated our results comprehensively, by utilizing 200 ns of MD simulation approach. RMSD, RMSF, Rg, and B‐factor results showed the decrease the stability and interactions in all the mutants, as compared with wild type. Herein, it is important to emphasize that similar multistep computational pipelines, combining prediction algorithms, structural modeling, and MD simulations, have been effectively employed in other biological systems to validate functional impacts of mutations. For instance, Hoda et al. [69] utilized an integrated bioinformatics and MD‐based approach to study mercuric reductase from *Pseudomonas fluorescens*, revealing conformational mechanisms underlying mercury detoxification. Such studies reinforce the methodological rigor of combining sequence‐based predictions with dynamic validation, as adopted in our present work on *AGTR2* [69]. Unlike earlier studies that primarily rely on prediction tools without downstream validation, we combined functional, structural, dynamic, and interaction‐based analyses to comprehensively characterize *AGTR2* nsSNPs [70, 71]. Finally, we investigated the PTM site presence of the *AGTR2* protein at nsSNP locations. Remarkably, for the most detrimental nsSNP locations, neither phosphorylation nor ubiquitylation sites were identified. At nsSNP locations, 13 phosphorylation sites were found by GPS3.0 and NetPhos3.1. Based on GeneMANIA and STRING predictions, *AGTR2* was the most often interacting protein with HTR2B. AGT, EDN1, REN, COL1A1, AGTR1, TIMP3, ACE, AGTRAP, MTUS1, RXFP, PTPN6, and PIK3CB are just a few of the proteins that have been linked to cardiovascular diseases. This underscores the importance of the *AGTR2* protein in these circumstances [72]. Moreover, similar integrative strategies have recently been applied to other disease‐linked genes such as RET [73], TP53 [74], and BRCA1/2 [75], where structural modeling and MD simulations have explained how deleterious variants disrupt folding stability and protein–protein interfaces. These researches further strengthen the rationale of utilizing this comprehensive computational framework, specifically to *AGTR2*. Previous SNP‐focused studies typically centered on genes unrelated to cardiovascular function or did not assess real structural impact through MD simulation or protein interaction disruption [70, 71]. Our study is among the first to integrate these deeper validations specifically for *AGTR2*, providing a more biologically and clinically relevant understanding of its role in cardiovascular disorder. This study effectively highlights the role of potentially deleterious mutations within *AGTR2* gene in the onset of the various cardiovascular diseases. Our findings also underscore a broader methodological perspective that supports the integration of bioinformatics predictions with structure‐based and dynamic simulations, representing a growing and powerful trend in SNP and mutation research.

## 5. Conclusion

This insight illustrates how nsSNPs can affect the *AGTR2* protein′s structure and functionality. Out of 1777 nsSNPs, 18 were predicted deleterious using six widely used nsSNPs prediction tools. These nsSNPs were further validated for their potential to induce any structural and functional alteration, and stability change to the *AGTR2* structure, followed by their conservation and topological analyses, which resulted in the suggestion of four most damaging nsSNPs. These four nsSNPs, including I135N (rs1556673736), P271L (rs3729979), W269C (rs15566673810), and I132F (rs200599388), were evaluated using secondary and tertiary structure analyses. Furthermore, validation of their damaging effects using molecular docking and MDs simulation strengthened our research. Overall, these nsSNPs were considered to be crucial in the emergence of cardiovascular disorders brought on by dysfunctions of *AGTR2*, opening the door to precision medicine and the discovery of workable remedies. Any laboratory assessment will provide more in‐depth knowledge about our proposed nsSNPs.

## Author Contributions

M.W.I. and M.S.: data collection, methodology, software, results, writing—original draft; M.B., S.A., X.S., G.A.S., and G.Z.: validation and writing—review & editing; Q.Y. and M.D.: supervised the project, funding acquisition, approved, and submitted the final manuscript. M.W.I. and M.S. contributed to the paper equally.

## Funding

This study was supported by National Key R&D Program of China (2021YFC2101503, 2021YFC2102900); Beijing Natural Science Foundation (L212001); King Saud University, Riyadh, Saudi Arabia , through the Ongoing Research Funding Program (ORF‐2026‐1118); and State Key Laboratory of Chemical Resources Engineering, Beijing University of Chemical Technology, Beijing 100029, China.

## Disclosure

None of the mentioned funding sources had any role in the study design, or any relevant tasks.

## Ethics Statement

Ethical approval is not applicable to this article. This article does not contain any studies with human or animal subjects.

## Consent

There are no human subjects in this article and informed consent is not applicable.

## Conflicts of Interest

The authors declare no conflicts of interest.

## Supporting information


**Supporting Information** Additional supporting information can be found online in the Supporting Information section. The specific command‐line inputs used for PCA and FEL analyses are documented in Supporting Material S1.

## Data Availability

Data are available from the corresponding authors upon reasonable request.
