# Supplementary material for: Delving Into the Depths of AGTR2: In Silico Identification of Deleterious Nonsynonymous SNPs Associated With Cardiovascular Diseases
Source: Hum Mutat. 2026 Apr 15;2026:9394808. doi: 10.1155/humu/9394808 (PMC13084195; doi:10.1155/humu/9394808)
Supplement: Supplementary file 1 — Supporting Information Additional supporting information can be found online in the Supporting Information section. The specific command‐line inputs used for PCA and FEL analyses are documented in Supporting Material S1. [file HUMU-2026-9394808-s001.pdf]

Supplementary Material S1

Specific Commands

Principal Component Analysis (PCA)

Script name: PCA.in

Script:

```
parm complex_solvated.top
trajin prod.crd
rms first :1-978&!@H=
average crdset 3SCI-average
createcrd 3SCI-trajectories
run
crdaction 3SCI-trajectories rms ref 3SCI-average :1-978&!@H=
crdaction 3SCI-trajectories matrix covar name 3SCI-covar :1-978&!@H=
runanalysis diagmatrix 3SCI-covar out 3SCI-evecs.dat vecs 10 name myEvecs nmwiz nmwizvecs 10 nmwizfile 3SCI.nmd nmwizmask :1-170&!@H=
crdaction 3SCI-trajectories projection Mode modes myEvecs out projection.txt beg 1 end 10 :1-978&!@H= crdframes 1,last
hist Mode:1 bins 50 out 3SCI-hist.agr norm name Mode-1
hist Mode:2 bins 50 out 3SCI-hist.agr norm name Mode-2
hist Mode:3 bins 50 out 3SCI-hist.agr norm name Mode-3
hist Mode:1 Mode:2 bins 50 out hists_1-2.gnu name PC12 free 300
hist Mode:1 Mode:2 bins 50 out hists_1-3.gnu name PC13 free 300
hist Mode:1 Mode:2 bins 50 out hists_2-3.gnu name PC23 free 300
run
clear all
readdata 3SCI-evecs.dat name Evecs
parm complex.top
parmstrip !(:1-978&!@H=)
parmwrite out 3SCI-modes.prmtop
```

```
runanalysis modes name Evecs trajout 3SCI-mode1.nc pcmin -100 pcmax 100 tmode 1 trajoutmask :1-978&!@H= trajoutfmt netcdf
```

Terminal Commad: cpptraj -i pca.in

Free Energy Landscape (FEL)

Script name: create-fel-MODES.sh

Script:

```
#!/bin/bash
read -p "Enter input names:" name1
MDINPUTS=($name1)
for input in ${MDINPUTS[@]}; do
	awk '$1 == "1", $1 == "20000" { print $1,$2}' ${input}_projection.txt > ${input}_MODE1.xvg
	awk '$1 == "1", $1 == "20000" { print $1,$3}' ${input}_projection.txt > ${input}_MODE2.xvg
done
```

Terminal Commad: chmod u+x create-fel-MODES.sh

Script name: FEL.sh

Script:

```
#!/bin/csh
echo "pearl Script Started"
perl sham.pl -i1 file_MODE1.xvg -i2 file_MODE2.xvg -data1 . 1 -DATA2 . 1 -o gsham_input.xvg
echo "GMX Script Started"
gmx sham -f gsham_input.xvg -ls Free-Energy-Landscape.xpm
echo "Python Script Started"
python3 xpm2txt.py -f Free-Energy-Landscape.xpm -o Free-Energy-Landscape.txt
echo "FEL DONE"
```

Terminal Commad: chmod u+x FEL.sh

Commands:

bash create-fel-MODES.sh

bash FEL.sh
